# Applicants as reviewers: a mixed methods evaluation of the risks, benefits, and potential of distributed peer review for grant funding allocations

**DOI:** 10.1186/s41073-026-00246-7

**Published:** 2026-09-12

**Authors:** Anna Butters, Melanie Benson Marshall, Stephen Pinfield, Tom Stafford, Alexander Bondarenko, Barbara Neubauer, Robert Nuske, Pierre Schwidlinski, Hanna Denecke

**Affiliations:** 1 School of Information, Journalism and Communication, University of Sheffield, The Wave, 2 Whitham Road, Sheffield, S10 2AH UK; 2 Research On Research Institute, London, UK; 3 School of Psychology, University of Sheffield, ICOSS Building, 219 Portobello, Sheffield, S1 4DP UK; 4 Volkswagen Foundation, Hannover, Germany

**Keywords:** Funding evaluation, Peer review, Distributed peer review, Research funding, Mixed methods

## Abstract

**Background:**

Peer review-based funding evaluation systems are under increasing pressure. To help alleviate this, innovative approaches like Distributed Peer Review (DPR) – where applicants review proposals for the same funding call – are being explored. The Volkswagen Foundation trialled DPR alongside a conventional panel review process for an interdisciplinary call. Evaluation of this trial provides insights into the dynamics of (distributed) peer review.

**Methods:**

All proposals (*N* = 140) underwent both DPR and the panel review process. In DPR, applicants (*N* = 323) reviewed four or five proposals, providing an overall score, scores for each funding criteria, and a comment. Proposals were ranked using a trimmed mean score. Expectation and feedback surveys were administered. The panel process involved shortlisting by VWS staff, review by panellists (*N* = 8), and a final panel discussion. Semi-structured interviews with applicants, panellists, and VWS staff (*N* = 20) explored perceptions and experiences of DPR, using thematic analysis. Quantitative analysis of review data and surveys utilised bootstrapping to assess decision stability and mixed effects models for consistency between reviewers. Qualitative and quantitative results were integrated in a convergent parallel design.

**Results:**

DPR recommended ten proposals for funding, while the panel recommended eleven; three overlapped. Funding criteria strongly predicted overall score; ‘Originality’ was given most weight by both sets of reviewers. Stability of DPR funding decisions was modest; across 500 bootstrapped samples a mean of 5.49 out of ten previously funded proposals would still be funded. This may be explained by high inter-reviewer variability: more variance was attributable to between-reviewer differences (20.0%) than between-proposal differences (8.9%). Advantages of DPR included increased efficiency, faster decision times, detailed feedback to applicants, and democratisation of funding evaluation. Noted disadvantages were burden on applicants, absence of discussion, and perceived lack of reviewer expertise. Funders implementing DPR must consider the high initial effort, potential for ‘gaming’, and reduced direct influence.

**Conclusions:**

This trial demonstrates the feasibility of DPR for an interdisciplinary funding programme. Implementing DPR requires funders to accept trade-offs and that DPR may not produce the same outcomes as existing systems. Building on established peer review principles, it may offer benefits, including shortening time to funding decision, and has the potential to contribute to the democratisation of funding evaluation.

**Supplementary Information:**

The online version contains supplementary material available at https://doi.org/10.1186/s41073-026-00246-7.

## Background

Peer review is ubiquitous across research systems, including funding evaluation. It is generally accepted by the research community [1, 2] and considered necessary to maintain the autonomy of science [3]. However, it faces criticism for limited reviewer agreement [4, 5], biases disadvantaging particular applicant groups [6–8], conservatism [9–11], and lack of transparency [12, 13]. The significant burden on all stakeholders [3, 12, 14] also makes evaluation costly and, sometimes, inefficient and time consuming [15, 16], with recruitment of suitable reviewers proving increasingly challenging [17, 18]. Reducing this burden is essential and motivates innovative approaches to evaluation [1, 19].

One such innovation is Distributed Peer Review (DPR), where applicants also serve as reviewers for other submissions to the same call. This immediately addresses reviewer recruitment challenges. Initially proposed to ease review processes for telescope time allocation [20], it has been successfully adopted in astronomy [21–24] and used widely for evaluating computer science conference papers [25]. Several research funders have also trialled this approach [26–30].

Previous DPR trials demonstrate diverse implementations, highlighting both concerns and cause for optimism. Much existing evidence comes from evaluation of DPR’s use in the astronomy community. A 2018 experiment at the European Southern Observatory found machine-learning-enhanced DPR was well-received and no more subjective than standard peer review [22, 31]. At the ALMA observatory, most reviewer comments to applicants were rated helpful; quality of feedback was consistent across career stages [23, 24]. A second stage allowing reviewers to see others' ranks/comments resulted in only 15% changing their initial feedback. Evidence from funding organisations is more limited. The US National Science Foundation reported DPR was more efficient and produced more comprehensive reviews, but its bonus points system raised concerns about bias towards consensus [26, 32]. Recently, UKRI’s [28] trial of DPR for allocating Early Career Researcher (ECR) AI fellowships reduced time-to-decision by 53%–65%.

DPR has potential to address critical pain points in grant evaluation [28, 33]. Reviewer recruitment is streamlined, providing a topic-aligned reviewer pool that grows with applications, making the process scalable and potentially reducing time-to-grant [24, 26, 28, 33]. It may deter speculative applications and provide applicants with more diverse, comprehensive feedback [24, 31, 33]. Reviewing can also help applicants better understand criteria, increase reviewer diversity, and provide valuable training in grant reviewing and writing [22, 28, 31].

However, concerns remain, most significantly the potential for ‘gaming’ (strategic scoring to boost one’s own chances) [25, 28, 33], which designers of DPR trials actively try to prevent (e.g., [27, 28]). Issues of confidentiality and risk of plagiarism or 'scooping' are also highlighted [20, 31]. Other concerns include increased applicant workload and limited reviewer expertise, since reviewing is restricted to applicants [28, 33]. Finally, DPR remains susceptible to some of the same criticisms as conventional peer review, including bias.

We report on a 2024 experiment by the Volkswagen Foundation (VWS), a German private funder, which trialled DPR in parallel with a conventional panel review process for their interdisciplinary, innovation-focussed, “Open Up” programme. We present a mixed-methods evaluation aiming to contribute to the limited evidence regarding use of DPR in funding evaluation, offer insights into the (distributed) peer review process, and provide guidance for funders considering this approach. We address the following research questions:

- RQ1 What were different stakeholders' expectations for the DPR experiment?
- RQ2 What are the main points of comparison between DPR and the classic panel process?
- RQ3 How reliable/robust is the evaluation conducted using DPR?
- RQ4 How did different stakeholders perceive DPR after seeing it in practice?
- RQ5 When and where is DPR most, and least, suitable for use?
- RQ6 What are the key management challenges in managing a DPR process?

Although our evaluation is able to address these aspects of DPR it is beyond the current scope to answer questions with respect to the value of projects selected. Reasons for this include the relatively low sample of funded proposals and the lack of elapsed time since selection for the value of the projects selected by both DPR and the traditional method to be realised.

## Methods

### Design

We used a mixed-methods design (convergent parallel), integrating survey, review, and qualitative interview data to comprehensively address the research questions [34]. Although primarily convergent, preliminary findings from each data stream informed the other (e.g., quantitative results shaped the interview guide, and qualitative findings suggested further quantitative analysis). The study is reported according to the APA Journal Article Reporting Standards for Mixed Methods Research (JARS – Mixed).

### Setting

The Volkswagen Foundation’s ‘Open Up’ funding call focuses on opening up new research spaces in the humanities and social sciences. Projects submitted to this call are expected to take an interdisciplinary approach to exploring new and complex research spaces or topics.

**Fig. 1:**
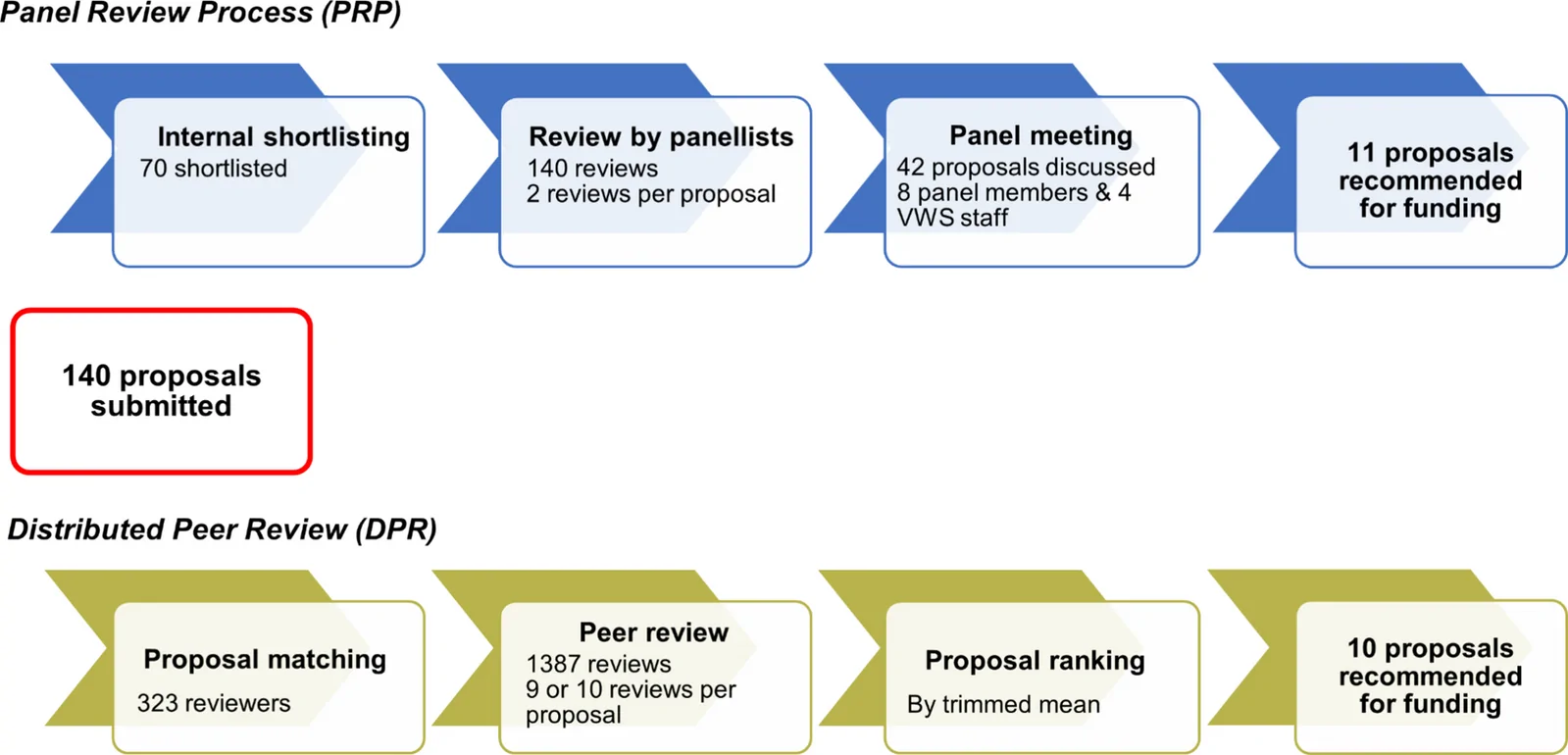
Panel review and DPR processes as implemented at VWS. Further details of both processes are available in [35]

For the 2024 ‘Open Up’ funding call (*N* = 140 submissions), proposals underwent both the panel review process and DPR using a double-blind process (Fig. 1).

#### Panel review process

- Shortlisting: VWS programme directors shortlisted submissions, removing ~50% per discipline
- Review: Shortlisted proposals reviewed by two panellists using the same criteria and template as DPR
- Discussion: 42 proposals (receiving at least one ‘A’ grade) discussed during a panel meeting.

#### DPR

- Reviewer Assignment: Proposals assigned to applicant-reviewers by algorithm, avoiding within-institution matches and prioritising aligned disciplines
- Workload: Applicant-reviewers completed a mean of 4.36 reviews (SD = 0.54) each
- Proposal Evaluation: Each proposal received a mean of 9.92 reviews (SD = 0.28) (mean number within-discipline reviewers = 6.81, SD = 2.35; mean number outside-discipline reviewers = 3.69, SD = 2.31)
- Scoring: Highest and lowest scores for each proposal trimmed; remaining scores averaged to establish final proposal ranking.

Funding recommendations from both processes were ratified by the Foundation’s board, and decisions and DPR review comments shared with applicants. Comments were only shared with the applicants who submitted a proposal; those reviewing a proposal did not have access to other reviews for the same proposal.

### Participants

#### Quantitative

All applicants and co-applicants (Table 1; *N* = 323, minus two excused) for the 'Open Up' funding call were included and consented to serve as reviewers; thus, all participants were self-selected. No exclusion criteria were used. While there were no financial incentives, participants received written feedback (which they would not usually) and, with VWS committing to fund proposals identified via DPR and panel, may have been incentivised by slightly increased likelihood of funding.

**Table 1:** Characteristics of applicant-reviewers (*N* = 323*)

Characteristic	Count	Percentage
*Gender:*		
Diverse	3	0.9%
Female	151	46.8%
Male	160	49.5%
No answer	9	2.8%
*First generation:*		
Yes	114	35.3%
No	194	60.1%
I don't know	4	1.2%
No answer	11	3.4%
*Working years (quantiles):*		
0–8 yrs	89	27.6%
9–12 yrs	72	22.3%
13–19 yrs	80	24.8%
20–52 yrs	81	25.1%
No answer	1	0.3%
	**Mean (SD)**	**Median (IQR)**
Working years	14.47 (8.90)	12.5 (11.75)

^*^Two applicants were excused due to personal circumstances

#### Qualitative

Applicants, panel members, and VWS staff were invited by email to be interviewed about their attitudes towards and experience of the process. Twenty stakeholders participated in an interview: seven successful applicants, four unsuccessful applicants, five panel members, and four VWS staff. This sample enabled us to collate a variety of perspectives on the experiment. During the recruitment and consent process, all interview participants (applicants and panellists) were explicitly informed via written consent forms and verbal briefings that the evaluation was being conducted by an independent research team at the University of Sheffield in partnership with VWS. They were assured that their transcripts would be pseudonymised if requested, stored on secure university servers, and that no raw data or identifying information would be accessible to VWS management or affect future funding decisions.

### Researcher description

Evaluation was undertaken by a multidisciplinary research team with backgrounds in Information Science and Psychology, including two ECRs, one mid-career and one late-career researcher. Whilst members of the team specialising in qualitative or quantitative research were responsible for the respective strands, we met regularly to discuss progress, share results and consider potential interpretations.

### Data collection

**Fig. 2:**
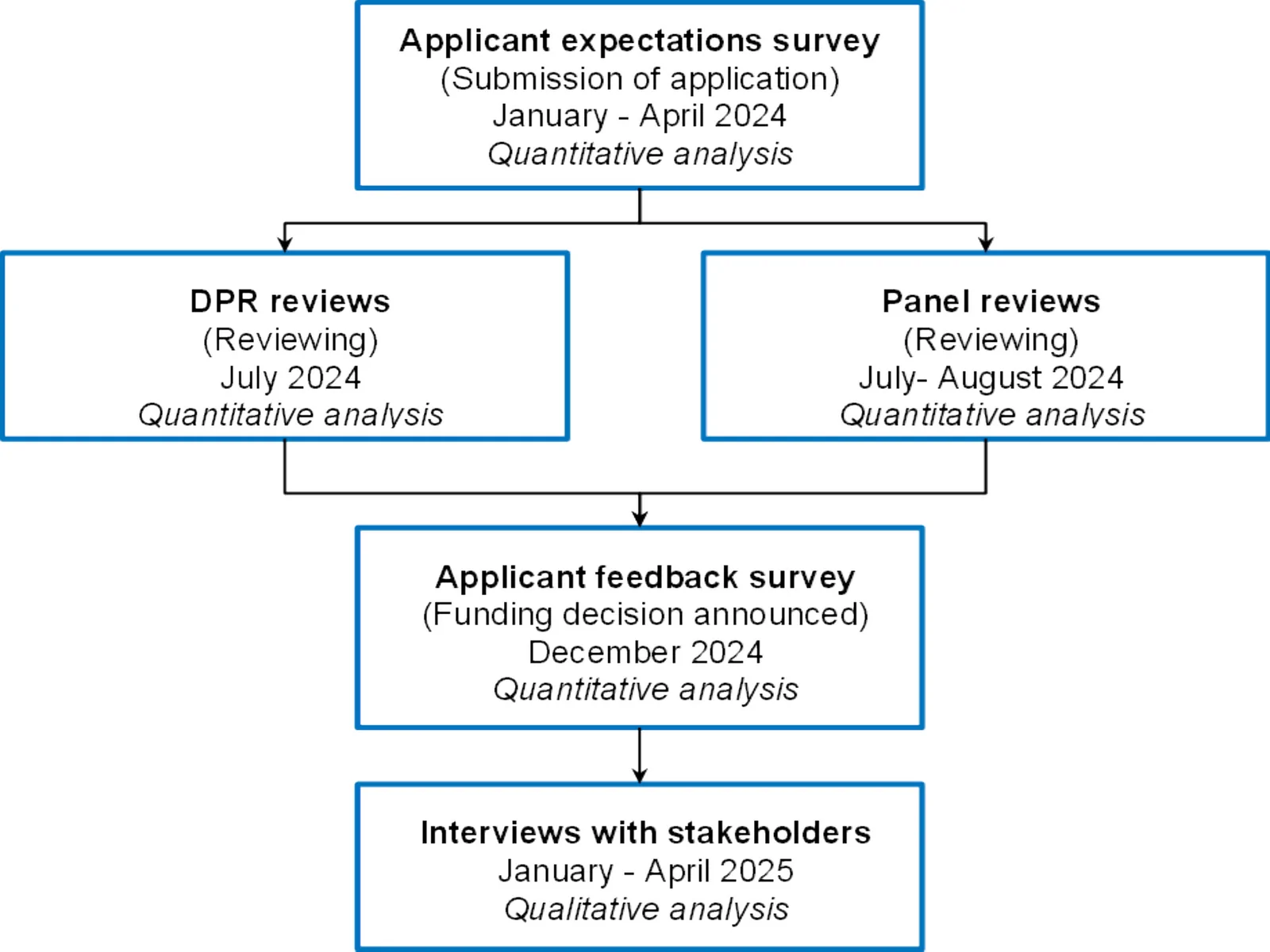
Workflow of datasets, point of data collection, date of data collection and type of analysis

Quantitative data [36] were collected at three stages: application, review, receipt of funding decision (Fig. 2).

Measures included at each stage are detailed in Table 2 (full measures at https://osf.io/cws8b/). Reviews were completed via LimeSurvey. All applicants provided data at application and review submission; an optional feedback survey following the funding decision was completed by 127 applicants (from 84 proposals). Funded applicants were more likely to respond relative to unfunded applicants; however, respondent characteristics were representative of the full applicant population (Supplementary Table 1). Data were also collected for each decision-making stage of the panel process: shortlisting, review, panel recommendation. Data were pseudonymised prior to sharing with researchers.

**Table 2:** Measures according to stage of data collection

Measure	Stage of data collection	Description
Disciplinary self-identification	Application	One to six subject areas (based on German Research Foundation classifications) which applicants felt competent to review in
Proposal discipline	Application	One to six subject areas which applicants felt were associated with their proposal
Expectations of DPR	Application	Extent of agreement (7pt scale, ‘strongly agree’ to ‘strongly disagree’) with six statements regarding DPR (e.g., ‘This process will identify appropriate reviewers’) and how it would compare to panel review (e.g., ‘I expect the process to select a similar set of proposals to panel review’)
Applicant characteristics	Review	Gender, whether they were the first generation of their family to attend university, and number of years working in research
Proposal score	Review*	Overall score awarded (9pt scale from A + (‘outstanding’) to C- (‘unsuitable’))
Criteria scores	Review*	Scores (A + to C-) awarded for each of four funding criteria (‘Exploratory nature and novelty’, ‘Added value of the constellation’, ‘Originality’, ‘Scientific quality’.)
Highest/lowest score considered	Review*	Highest and lowest score the reviewer considered awarding to each proposal (A + to C-)
Disciplinary fit	Review*	Confidence in reviewing a proposal according to the fit between own knowledge and experience and the proposal topic (7pt scale, ‘Totally confident’ to ‘Certain that I was inappropriate to provide a review’)
Estimate of average scores	Review*	Prediction of the average score (A + to C-) which would be awarded to a proposal by all reviewers
Review time	Review*	Time taken to complete each review (in minutes)
Impression of reviews	Feedback	Rating (7pt scale, ‘very good’ to ‘very poor’) for overall politeness, helpfulness, and expertise of the reviews received
Constructiveness of reviews	Feedback	Constructiveness of each individual review (5pt scale, ‘constructive’ to ‘non-constructive’)
Attitude to future DPR	Feedback	Attitude towards participating in future funding calls using DPR (5pt scale, ‘very positive’ to ‘very negative’)

^*^With the exception of applicant characteristics all review measures were also completed during review by panellists

Interviews [37] were conducted in English and lasted between 20–60 min. They were semi-structured, with a core list of questions for each group addressing participants’ expectations, workload, disciplinary fit, scoring, feedback, perceptions, and the future of DPR (https://osf.io/cws8b/). The format allowed for deeper exploration of relevant issues. Interviews were conducted and recorded via Google Meet. Recordings were transcribed using its transcription function, then manually checked and edited for accuracy. Prominent themes were noted after each session to complement thematic analysis (see below).

#### Variables

##### Trimmed mean

Proposal scores were converted to a numeric scale (A + = 9, C- = 1), highest and lowest score per proposal removed and mean calculated.

##### Rank

Proposals were ranked according to their trimmed mean (1–140, ties allowed).

##### Reviewer uncertainty

Lowest score considered was subtracted from highest score considered to give a measure of reviewer uncertainty (0–8).

##### Disciplinary overlap

Where primary reported discipline of applicant and proposal under review matched, applicants were considered ‘within discipline’; where they did not, they were ‘outside discipline’.

#### Data analysis

Analysis of quantitative data was conducted in RStudio using R 4.4.1 (code at https://osf.io/cws8b/). All analyses were exploratory. Items using the A + to C- scale were re-coded to a numeric scale (1 = C-, 9 = A +). Self-rated disciplinary fit was re-coded to a 7pt scale (1 = ‘Certain that I was inappropriate…’, 7 = ‘Totally confident’). Associations between variables were examined using linear regression models. Assumptions were assessed using diagnostic plots; where they were violated, robust SEs were obtained (transformation of variables did not improve model fit).

Bootstrapping was used to examine the stability of DPR, i.e. the extent to which we would obtain the same results if the process were repeated. 500 samples were obtained and, for each sample, trimmed means recalculated and proposals reranked. The percentage of proposals always, sometimes, or never funded across the 500 samples was calculated (as per [38]). Sensitivity analyses were conducted using more bootstrapped samples (*n* = 1000, 2000, 3000). Sampling was repeated, with the number of reviews per proposal varying from three to 20.

Quartile agreement matrices, inspired by the procedure in [22], were produced to examine agreement between DPR and panel reviewers. 500 samples of two reviewers' (one DPR, one panel) scores for shortlisted proposals (*N* = 70) were obtained. Trimmed means and rankings were calculated for each reviewer. Quartile agreement for each sample and the mean across all samples was calculated.

Mixed effects models were conducted using the *lme4* package [39] to examine inter-rater reliability. The final intercept only model was:

$${\gamma}_{ij}= \mu + {\alpha}_{i}+ {\delta}_{j}+ {\epsilon}_{ij}$$

where ${\gamma}_{ij}$ is the score awarded, μ is the average score across all proposals, ${\alpha}_{i}$ is the random effect of the proposal, ${\delta}_{j}$ is the random effect of the reviewer, and ${\epsilon}_{ij}$ the residual error. Intraclass Correlation Coefficient (ICC) was calculated for random effects.

Agreement in review constructiveness between co-applicants (N proposals = 43) was evaluated with weighted kappa. Quadratic weights were used to account for the distance between disagreements [40]. Mean weighted kappa of 41 proposals (two excluded due to lack of variance) was calculated.

A qualitative inductive content analysis of interview transcripts was performed using NVivo 1.7.1, employing thematic analysis approaches [41]. Given that this study was commissioned by VWS and includes Foundation staff as co-authors, structural safeguards were established to ensure analytical independence and prevent funder bias in the qualitative evaluation. The thematic analysis was structurally partitioned, with the primary coding and initial theme generation being conducted independently by one empirical meta-researcher with a background in information science (MBM), with no ties to VWS. VWS co-authors were excluded from the raw data coding phase and did not have access to qualitative transcripts, to preserve participant anonymity. Data familiarisation was achieved through re-reading transcripts and preliminary notetaking. MBM inductively generated initial codes using a line-by-line approach, encompassing both semantic and latent coding. These codes were validated by other non-funder academic co-authors (ABu, SP, TS) through independent review of the source material, producing 65 codes overall (Supplementary Table 2). Codes were sorted and collated, and themes refined. To enhance rigour and dependability, transcripts were independently reviewed by academic co-authors to discern emerging themes; all final codes and themes were then confirmed by the academic team. To manage subjective bias, a reflexive research log was maintained, and code meanings were continually cross-checked against quantitative survey data (convergent parallel design) to ensure qualitative themes accurately reflected stakeholder sentiments rather than researcher preconceptions. The broader team, including VWS co-authors, met only during final synthesis stages to discuss the operational implications of the findings and review the final manuscript text. VWS staff involvement was strictly limited to contextualising the operational/management findings (RQ6) and ensuring the accuracy of the trial's institutional workflow descriptions. They exerted no influence over the interpretation of applicant or panel criticisms of the DPR process.

## Results

### Expectations of DPR (RQ1)

**Fig. 3:**
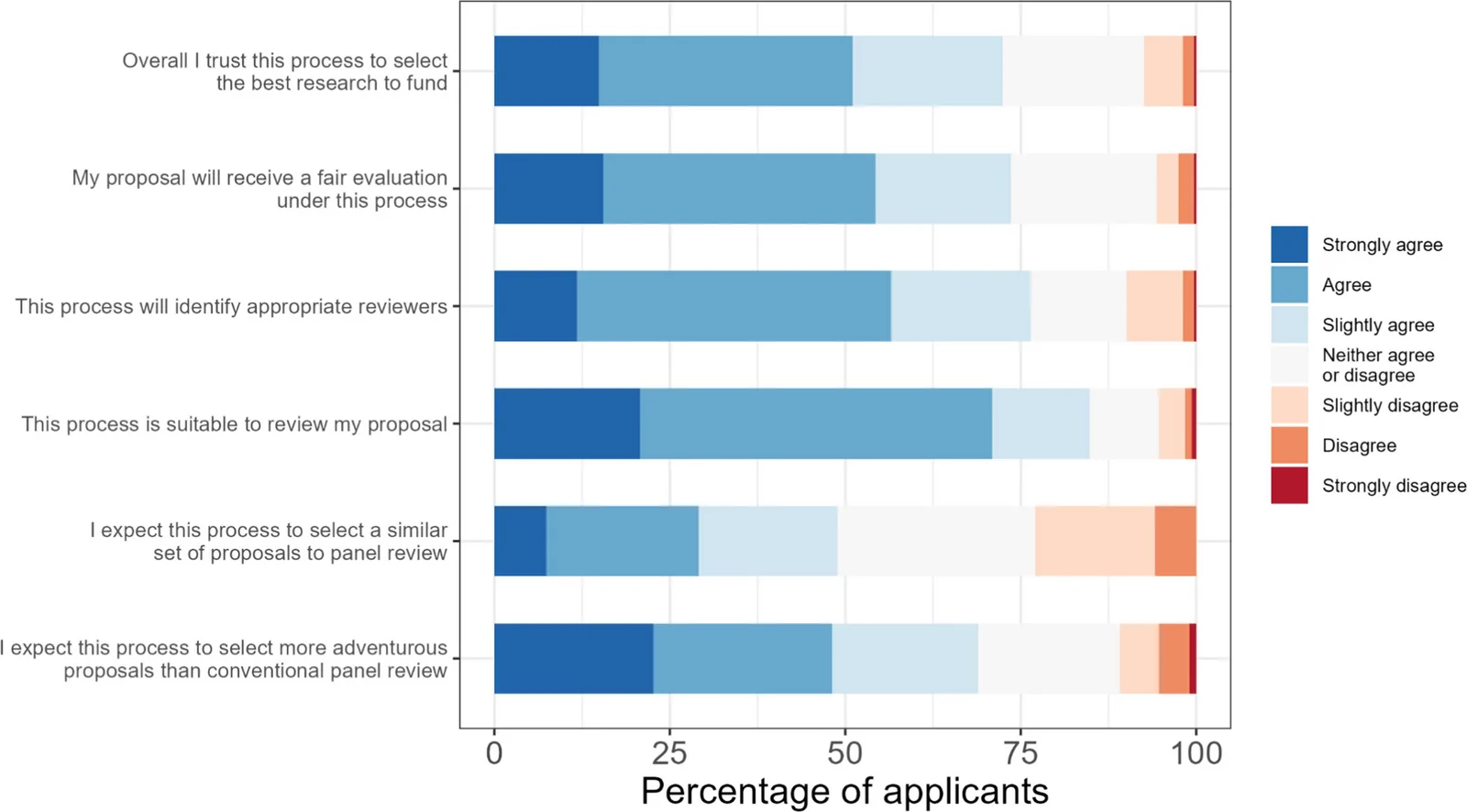
Applicant expectations of DPR and how DPR would compare to standard panel review process

Applicant expectations of DPR were generally positive (Fig. 3). Applicants were somewhat divided about anticipated overlap with panel decisions: less than 50% expected the two processes to select a similar set of proposals. Retrospectively, applicants cited reservations about the competitive nature of the process and the potential for “gaming”, alongside concerns about the significant time commitment and a perceived lack of reviewer expertise.

Panel members, while generally open and positive about integrating DPR, focused on review quality, emphasising the critical role of the discussion component in panel meetings for robust evaluation. They also shared the applicants' concern regarding the increased workload. VWS staff echoed concerns of both groups, with their main worry being that the increased burden on applicants could reduce application rates and harm the foundation's reputation. They, too, questioned whether the new process could maintain high standards of review quality and expertise.

### Comparison of DPR and panel review process (RQ2)

#### Comparison of funding decisions

**Fig. 4:**
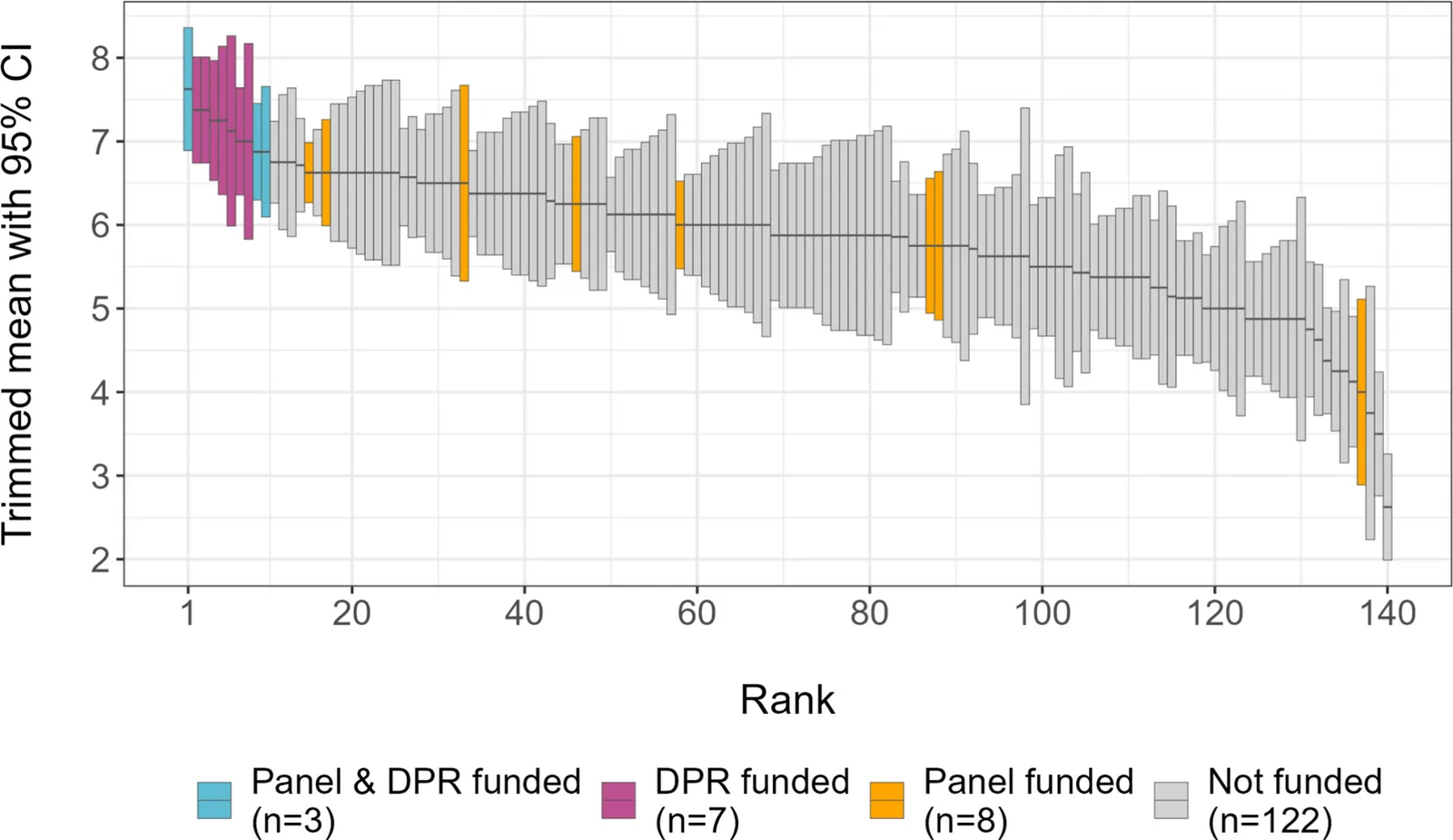
Trimmed mean of all proposals according to rank under DPR and funding status. N.B. For the purpose of this visualisation, when ranks were tied proposals were ordered by standard error. Any remaining ties were broken by proposal order

**Fig. 5:**
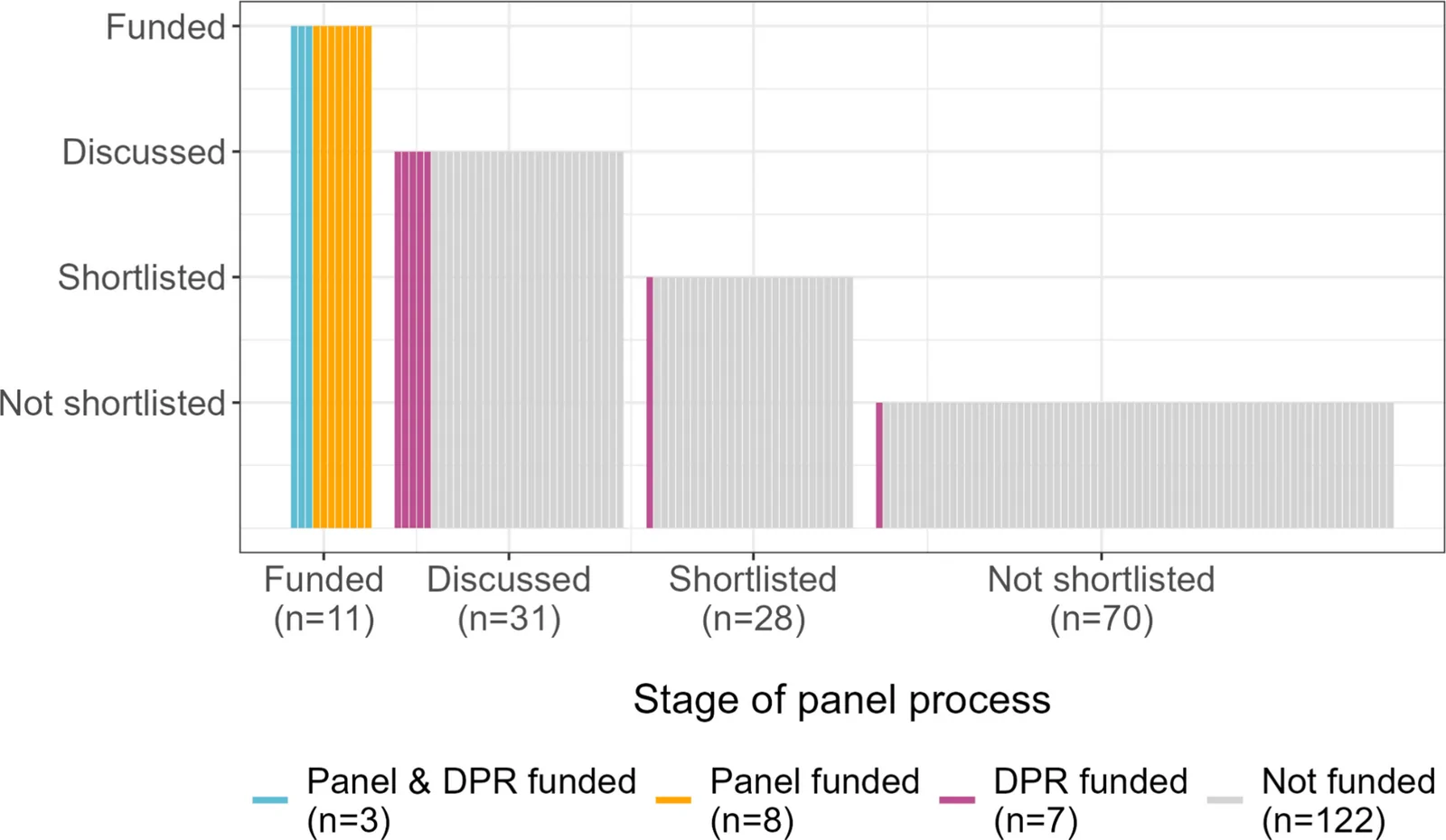
Proposal progression under panel review process and funding status under panel review process and DPR. Progression under panel review process according to furthest stage reached (shortlisted, discussed or funded)

Ten proposals were recommended for funding by DPR and 11 by the panel review process; three were selected in both processes. Panel-selected proposals were found across the full range of DPR ranks (Fig. 4) and DPR selected proposals across all panel review process stages (Fig. 5).

**Fig. 6:**
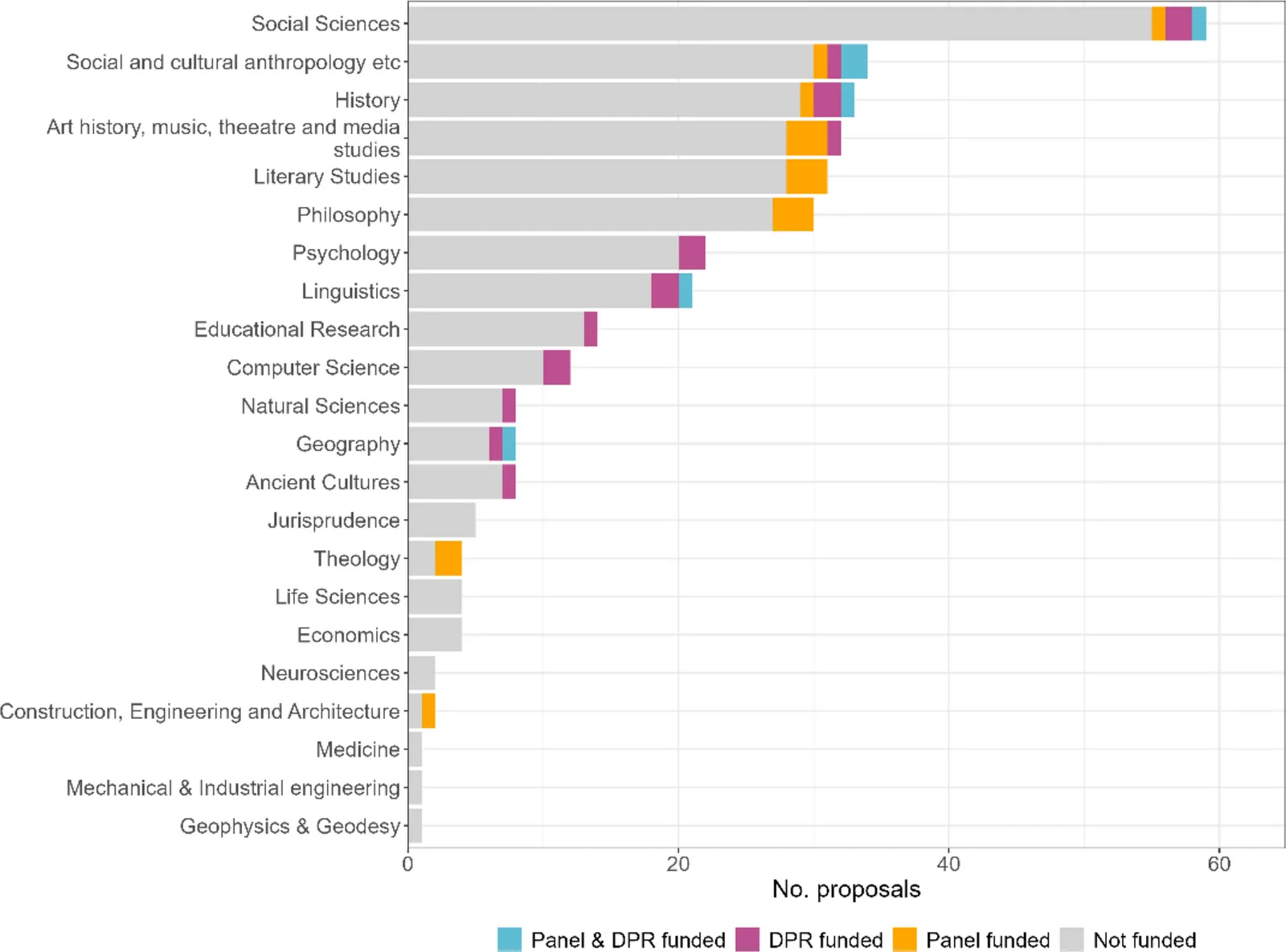
All reported disciplines of submitted proposals according to final funding status. One to six disciplines were selected for each submission. Disciplines are primarily reported at the level of review board (according to DFG classifications) and in a small number of cases at the level of research area (Mechanical & Industrial Engineering) or scientific discipline (life sciences, natural sciences)

Nine out of 10 of DPR funded proposals were shortlisted for assessment by panel reviewers, with eight out of 10 discussed during the panel meeting. Proposals funded by both processes spanned a range of disciplines (Fig. 6). A total of 22 disciplines (11 unique) were reported for DPR funded proposals and 21 disciplines (10 unique) were reported for panel selected proposals.

**Fig. 7:**
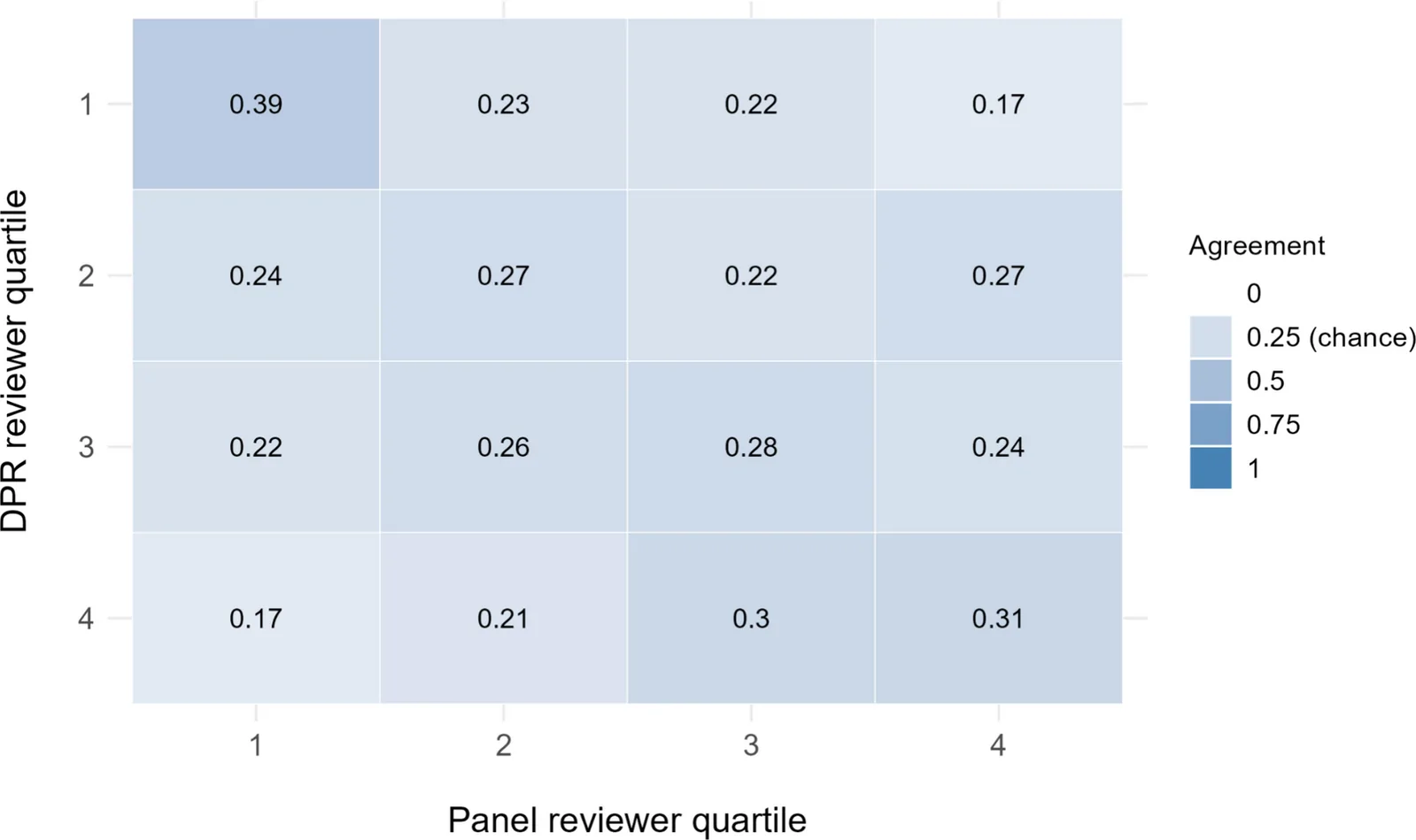
Quartile agreement matrix showing average proportion of agreement between a DPR reviewer and panel reviewer. Using scores from 500 bootstrapped samples for the 70 proposals which were internally shortlisted. Perfect agreement would produce scores of 1 on the diagonal and 0 s in other cells. Baseline agreement by chance alone would be 0.25 in all cells

Simulated rankings revealed modest agreement between DPR and panel reviewers (Fig. 7). Greater agreement in the first quartile (top 18) suggests ‘standout’ proposals were identifiable in both processes. Agreement was comparable with a previous evaluation of DPR [22]; indeed, agreement in the first quartile exceeded that reported in this previous study (0.31).

#### Comparison of scoring decisions

Average proposal and criteria scores were very similar between DPR and panel reviews where comparison was possible (Table 3), though DPR included all 140 proposals while the panellists reviewed only the 70 shortlisted proposals.

**Table 3:** Average criteria scores, overall score, lowest and highest scores considered, estimated scores and duration of reviewing from DPR and review by panellists

	**DPR reviews** (*N* = 1387)*				**Panellist reviews** (*N* = 140)*			
**Variable**	**Mean**	**SD**	**Median**	**IQR**	**Mean**	**SD**	**Median**	**IQR**
Proposal score	5.81	1.81	6	2	5.91	1.77	6	3
*Funding criteria scores*:								
Exploratory nature & novelty	6.03	1.95	6	3	6.18	1.73	6	3
Added value of the constellation	6.15	1.91	7	3	6.44	1.82	7	3
Originality	5.67	1.96	6	3	5.90	1.95	6	2.25
Scientific quality	5.96	1.95	6	3	6.06	1.74	6	3
Lowest score considered	4.63	1.92	5	3	4.36	1.78	4	3
Highest score considered	6.67	1.70	7	2	6.77	1.63	7	3
Estimated average score	5.79	1.68	6	2	5.71	1.55	6	2
Time per review (mins)	76.30	57.80	60	50	38.60	14.80	35	15
Total time per reviewer (hours)	5.56	4.20	4.00	4.17	11.18	3.06	9.92	5.17
Total review time (hours)	1763.10				279**			

^*^As DPR reviews include all 140 proposals and panellist reviews only the 70 shortlisted proposals, the resulting means are not calculated over identical sets of proposals

^**^Shortlisting, peer review and panel meeting

**Fig. 8:**
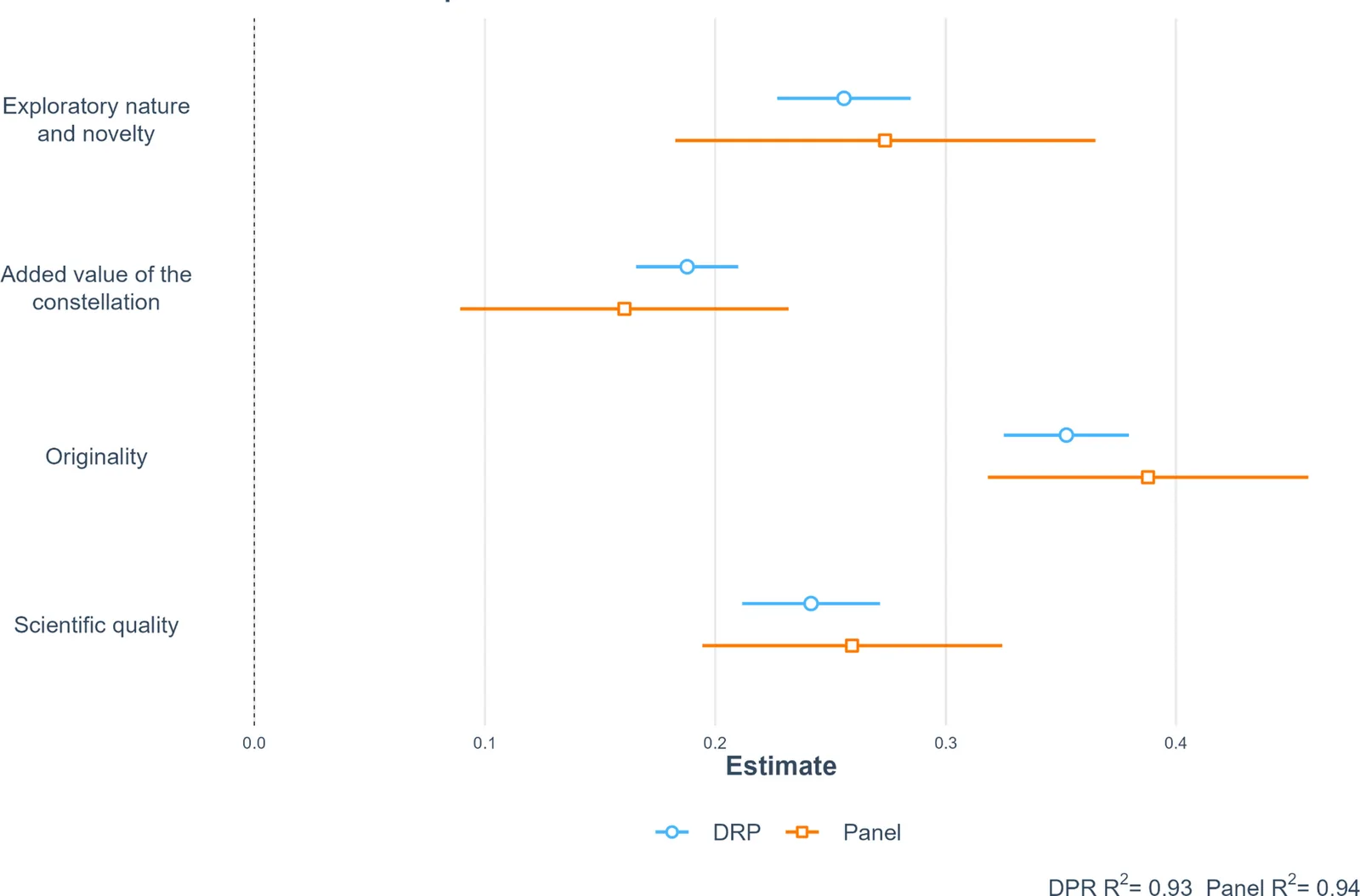
Regression coefficients for models evaluating association between scores for funding criteria and overall score. DPR (*N* = 1387) and panellist reviews (*N* = 140) evaluated in separate models

Both processes applied funding criteria consistently, with criteria scores highly predictive of overall score (Fig. 8). In both methods, “Originality” was given most weight, while “Added value of the constellation” was given least. However, interview data suggest a slight difference in focus. The panellist reviewers may have placed more weight on a proposal's overall quality, including its feasibility and methodology, while DPR reviewers might have been more influenced by the core concept's originality. Nevertheless, these findings suggest DPR and panellist reviewers were not only using criteria to inform the overall score but were also applying criteria in similar ways.

#### Nature of decision-making

A key difference between the two systems is the nature of the decision-making process. As one panel member stated, “Within the peer reviewing it is an isolated individual decision, whereas within the panel for me it was very obvious that it is a social decision, really affected by social human aspects” (P2). DPR relies on individual reviewer judgements, whereas panel review is a collaborative, social process, involving group discussions and interpersonal dynamics. While many panel members valued this collaborative aspect for addressing concerns and gaining new perspectives, they also acknowledged that factors like dominant personalities and waning motivation throughout the day could compromise impartiality. As one panel member observed, “If there's one difficult person it can really make an entire panel fail in their neutrality or fairness” (P13).

### Robustness of DPR (RQ3)

Reviewers reported some uncertainty (mean = 2.04, SD = 1.19) for most review decisions (Supplementary Fig. 1). Greater uncertainty was associated with awarding a lower score (F(1, 1385) = 33.48, *p* < 0.001, R^2^ = 0.02), though practically this was a small effect (ꞵ = −0.22, 95% CI[−0.30, −0.15]). Impact on a proposal's final rank appeared limited, with no significant association between average uncertainty per proposal and rank (F(1,138) = 2.93,* p* = 0.089, R^2^ = 0.02; ꞵ = 15.68 (95% CI[−2.42, 33.78]).

**Fig. 9:**
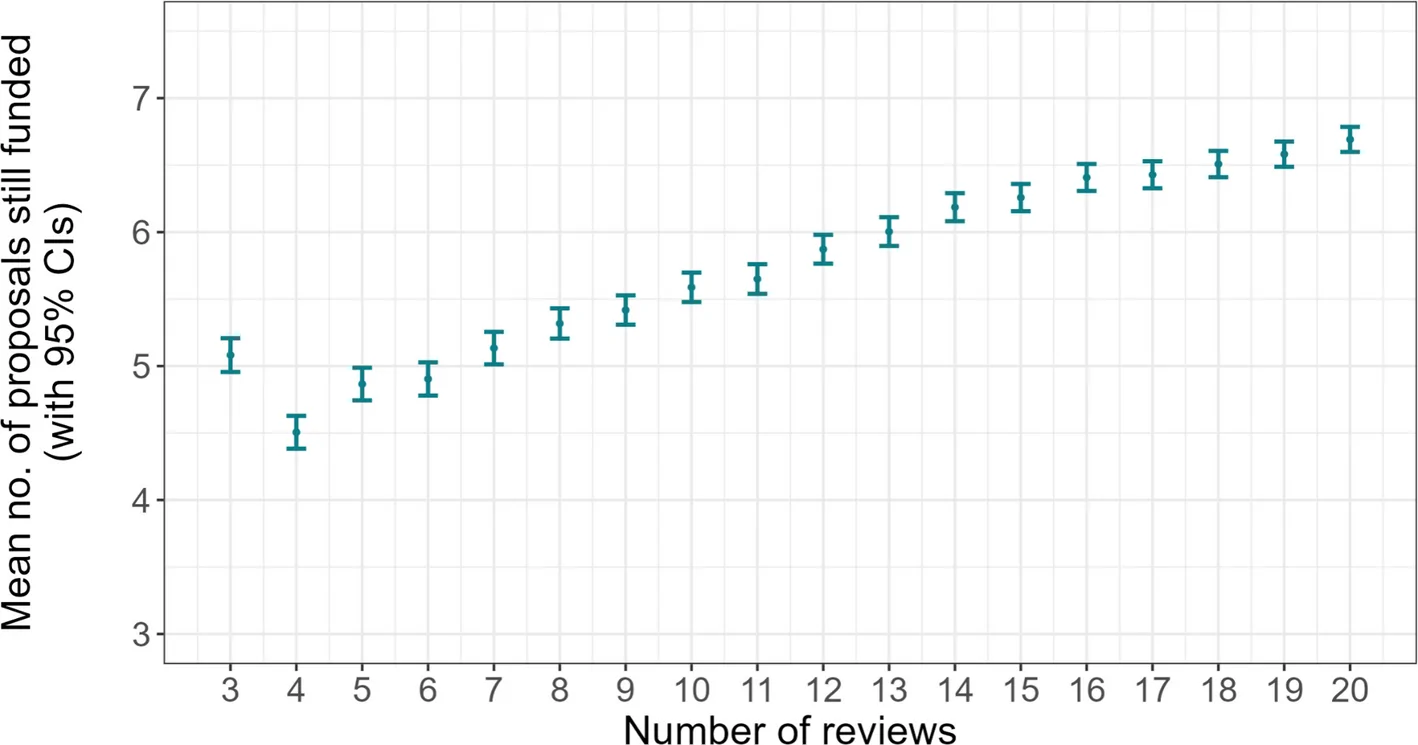
Mean number of DPR funded proposals still funded according to number of reviews per proposal. Across 500 bootstrapped samples for each possible number of reviews. Three reviews per proposal is an outlier due to use of trimmed mean. Trimming the highest and lowest score leaves one remaining score and as such averaging is not required

Stability was modest: across 500 samples, a mean of 5.49 (SD = 1.29) out of 10 originally DPR funded proposals were still funded. Across all proposals, 74.3% were sometimes funded and 25.7% were never funded. No proposal was always funded. Results were consistent across sensitivity analyses varying the number of bootstrapped samples (Supplementary Table 3). Increasing the number of reviews per proposal increased the mean number of originally DPR funded proposals which would be funded (Fig. 9). However, increases were gradual, with no obvious inflection point indicating a stage at which increasing the number of reviews ceased to improve stability.

Low inter-reviewer consistency may explain modest stability of funding decisions. Mixed effects models examined the effects of 1) proposal, 2) proposal and reviewer, and 3) proposal and reviewer with reviewer effects allowed to vary according to disciplinary overlap. Model two (Table 4) was the best fit (Supplementary Table 4). Inter-reviewer variability was relatively high: ICC indicated more variation in scoring was attributable to differences between reviewers (20.0% of variance) than to differences between proposals (8.9% of variance) when all reviews were included. When trimmed reviews were excluded this was reversed such that more variation in scoring was attributed to differences between proposals (19.1%) than to differences between reviewers (9.2%). As we might expect, removing extreme scores therefore appears to reduce, though not remove, inter-reviewer variability.

**Table 4:** Mixed effects model of the effects of reviewers and proposals on proposal score

	**All reviews** **(*N* = 1387)**						**Excluding trimmed reviews** **(*N* = 1107)**					
	**Fixed Effects**						**Fixed Effects**					
	β	**SE**	**95% CI**	**t**	***p***	**df**	β	**SE**	**95% CI**	**t**	***p***	**df**
Intercept	5.81	0.08	5.66, 5.96	76.32	<.001	208.88	5.86	0.07	5.72, 6.00	82.48	<.001	159.66
	**Random Effects**						**Random Effects**					
			**Variance**		**SD**				**Variance**		**SD**	
Reviewer (intercept)			0.66		0.81				0.20		0.45	
Proposal (intercept)			0.29		0.54				0.42		0.65	

Fixed effects t-tests use Satterthwaite’s method

Model equation: Score ~1 + (1|proposal) + (1|reviewer)

Reported as per best practice guidelines in [42]

Despite low inter-reviewer consistency, reviewers appeared to behave consistently *within* reviews. Scores for funding criteria strongly predicted overall score awarded (Fig. 8) indicating systematic application of the criteria.

### Stakeholder experience of DPR (RQ4)

#### Perceived advantages of DPR

A key advantage of DPR is its significantly shorter overall decision time; DPR reviewing occurred consecutively (~6 weeks) whilst panel activities were undertaken sequentially (~13–14 weeks). This efficiency allows for quicker allocation of funds and helps applicants start research or seek alternative funding sooner.

While DPR involved more total review hours (Table 3), this was shared among 323 applicants, meaning panel members (*N* = 8) each spent almost 2.5 times longer completing peer reviews, despite the total time in the panel process being substantially shorter. Panel members were faster at reviewing individual proposals, potentially due to greater experience, or possibly as a necessity due to heavy workload. They may also have anticipated further discussion at the meeting stage.

Applicants appreciated the capacity of DPR to offer more, and more diverse, feedback; a larger pool of reviewers can provide a wider range of critiques and suggestions, as well as giving each proposal greater attention. Under DPR, the median total review time for a single proposal was 12.33 h, significantly higher than the 45 min to four hours (depending on the stage reached) in the panel process.

DPR effectively solves the major hurdle of reviewer recruitment by utilising the ready-made applicant pool. While this reduced the coordination burden for the Volkswagen Foundation, staff still had to manage new procedures and a larger number of contacts.

Participating in the review process was seen as a valuable learning experience, especially for ECRs. It provided insights into strong submissions and funder expectations, which might in turn enhance proposal writing skills – a benefit for both applicants and funders.

Some interviewees felt DPR reviewers were more ‘true’ peers, fostering a “communitarian” spirit (P10), and generating a greater sense of investment and shared responsibility. Several participants also found the process intellectually stimulating, enjoying the diverse work and gaining fresh perspectives on their own research. This sense of innovation was generally seen as exciting, potentially attracting more participants, and did not deter applicants as VWS staff had initially feared.

Finally, a powerful advantage noted by interviewees is the perception that DPR is fairer and more democratic. Unlike traditional panels, where decisions can be swayed by group dynamics and bias, DPR distributes review responsibility broadly and anonymously, leading to decisions that may be seen as more objective and equitable, building greater trust and legitimacy.

#### Perceived challenges of DPR

Despite its advantages, DPR also presents challenges, such as increased burden on applicants. While most applicants interviewed found the increased workload manageable – partially due to the reduced proposal length for this call – the overall time and effort required must be considered for future implementations.

A persistent concern is the potential for “gaming.” A small number of applicants in this study awarded scores consistently and substantially below the proposal mean; these scores were generally excluded by trimming. However, because of the variety of possible strategic behaviours it is not possible to eliminate the possibility that some did occur. While conventional review can also involve bias, DPR’s direct competitive element requires strong ethical frameworks and oversight of strategies to reduce the likelihood of gaming and audit to review for potential occurrence. One strategy to mitigate gaming is to divide applications into pools and ask people to review only applications from a pool which does not contain their own submission (meaning their review cannot affect the likelihood of their own application being funded). This experiment did not use that approach because of the heterogeneity of applications. Where applications are more homogenous, applicants might reasonably be considered suitable reviewers for a greater proportion of submissions. In such circumstances, dividing applications into two or more pools is less likely to prove overly restrictive when matching proposals to reviewers.

DPR lacks the direct discussion and clarification found in traditional panel review processes, losing the opportunity for real-time problem solving and nuanced evaluation. However, this also avoids the negative influence of conformity biases and interpersonal dynamics seen in panel meetings.

There are concerns that DPR reviewers may be inexperienced or lack broad or deep expertise. Despite this, the mean self-rated disciplinary fit was statistically indistinguishable between groups (DPR = 4.92, SE = 0.03; Panel = 5.02, SE = 0.11). This suggests that while panel members may possess deeper expertise for *some* proposals, only a minority would likely benefit from the perceived advantage in disciplinary expertise attributed to panel members.

**Fig. 10:**
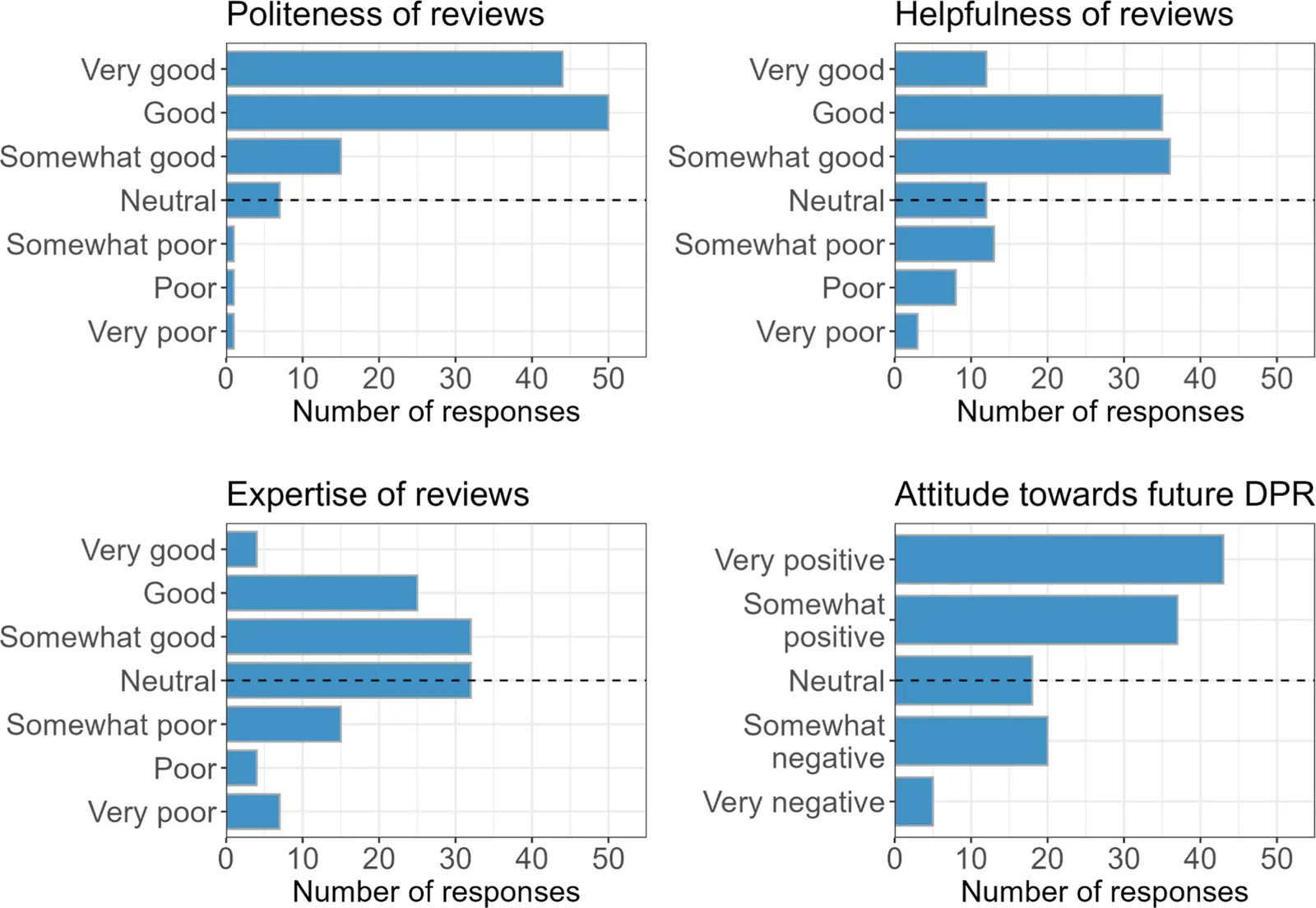
Applicant perceptions of the reviews they received and their attitude to future DPR. The helpfulness, politeness and expertise of the reviews received (*N* = 119) and attitudes to taking part in a future funding call using DPR (*N* = 123)

Many applicants found DPR reviews to be polite (91.6%) and helpful (69.7%) (Fig. 10).

**Fig. 11:**
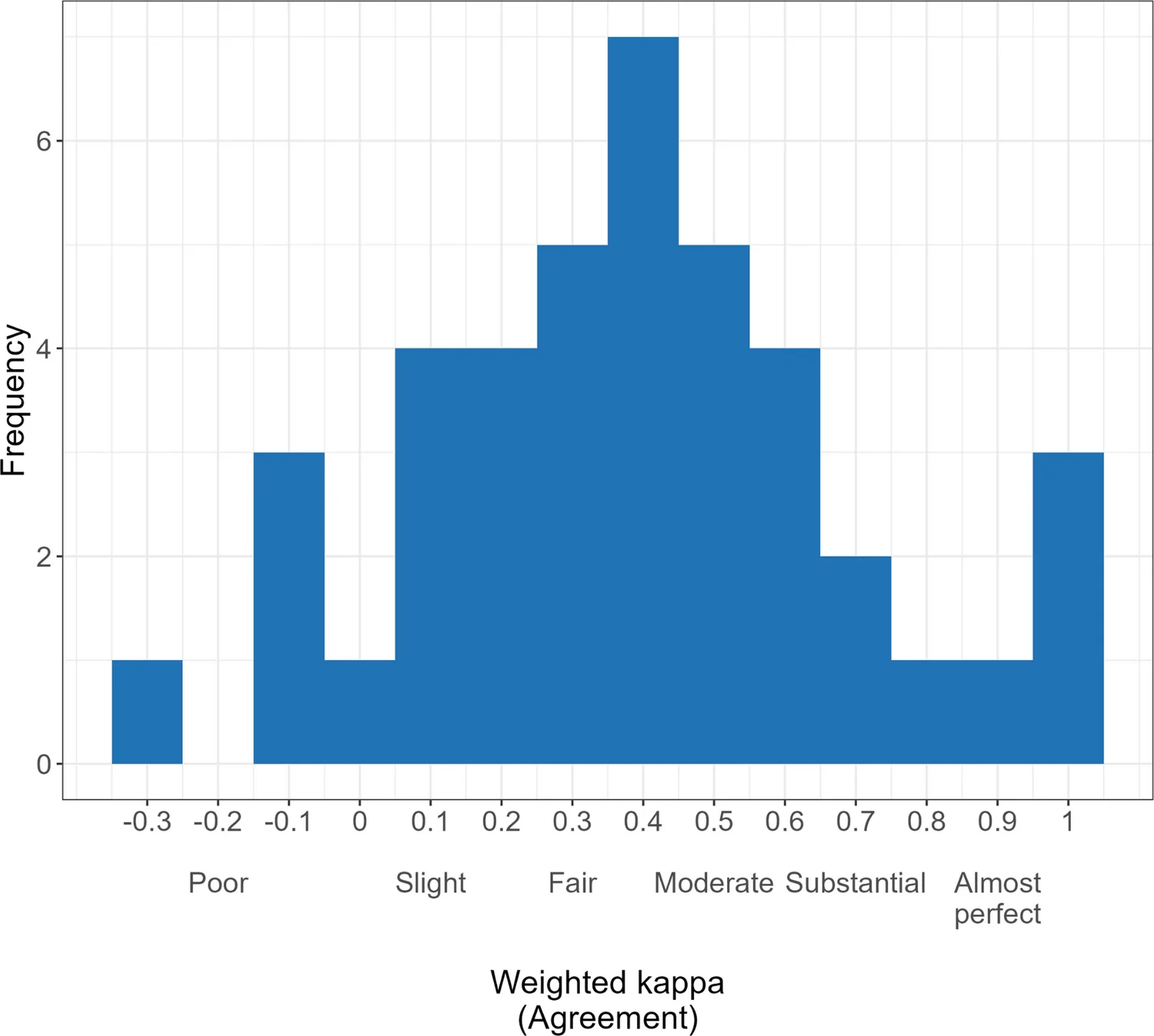
Distribution of weighted kappa (agreement) between co-applicants regarding the constructiveness of review comments. Interpreted according to guidelines in [43]

Opinions on reviewer expertise were more mixed. Initially, about three-quarters of applicants believed DPR would find appropriate reviewers, but post-participation, only 51% considered their received reviews’ expertise to be good. Most reviews were considered constructive (63.4%, Supplementary Table 5), but a substantial minority were also considered non-constructive (22.3%). Agreement on constructiveness among co-applicants on the same proposal was only fair (weighted κ = 0.38). This ranged from poor to almost perfect (Fig. 11), suggesting ‘constructiveness’ is subjective.

#### Attitudes towards future participation

**Fig. 12:**
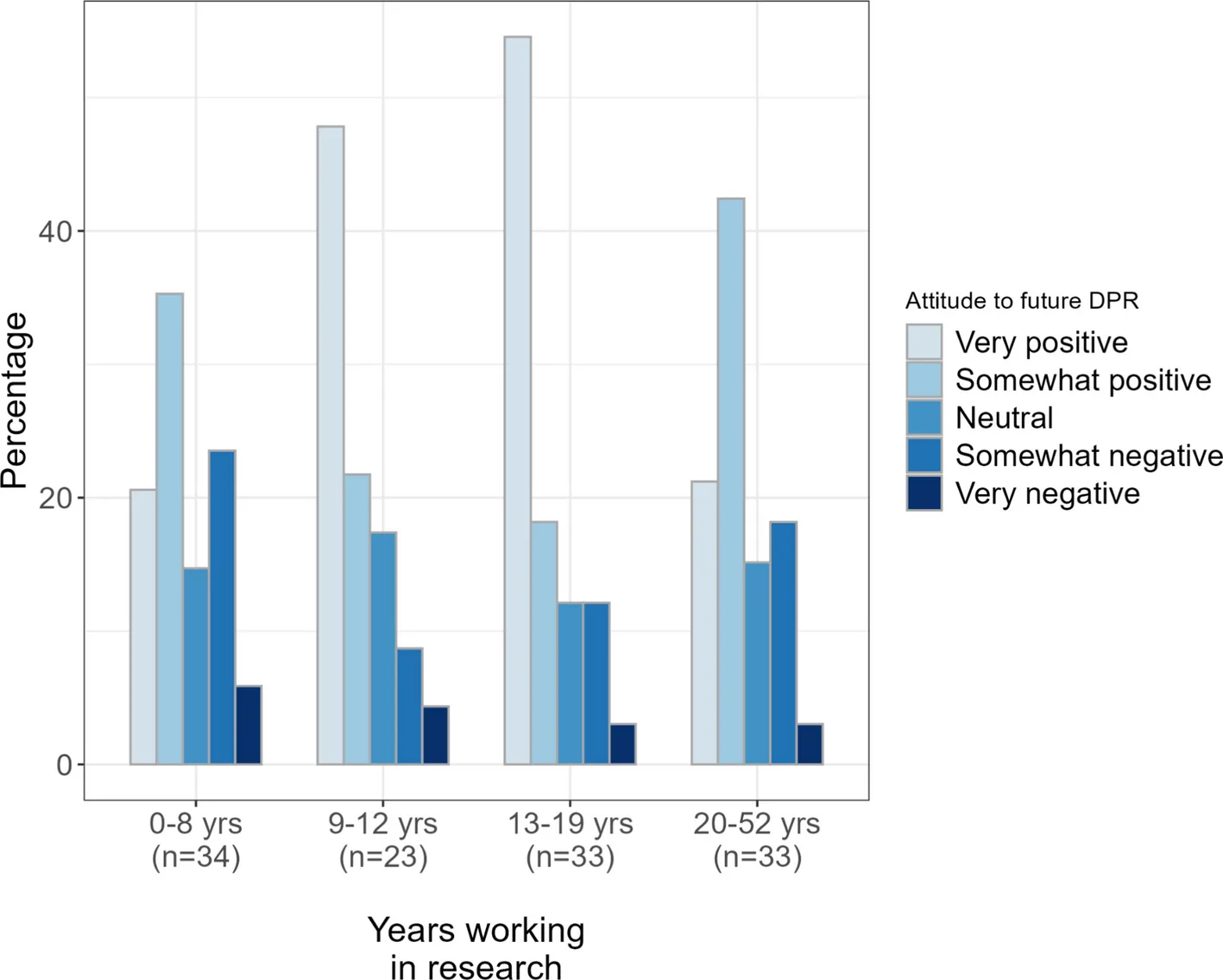
Attitude to future DPR according to years spent working in research

Both funded (82.8%) and unfunded (59.6%) applicants felt somewhat or very positive about future DPR participation, indicating a degree of acceptance. However, attitudes varied by career stage: early- and late-career researchers displayed more mixed feelings (Fig. 12). ECRs generally appreciated how DPR democratised the review process, providing experience typically reserved for senior academics, and insight into different fields and methods. They valued being reviewed by peers at a similar stage and felt empowered by the experience and the potential for reduced bias from established networks. However, ECRs also noted the increased workload and time commitment as a significant burden. Some worried about their own lack of expertise, fearing both giving and receiving poor quality feedback. One ECR described the process as “outsourcing” to non-expert reviewers (P12), which they felt undermined quality.

One predicted concern, theft of ideas, did not materialise. Interviewees felt that any appropriation would be easily traceable and difficult to execute, owing to the highly specialised subject matter of the projects and combined skillsets of the researchers. This may not apply to calls for more specific research areas.

### Suitability of DPR (RQ5)

Interviewees lacked consensus on the best type of funding calls for DPR. Some felt it was ideal for homogeneous calls (similar disciplines/topics), believing shared understanding would yield more insightful reviews. Others suggested it was beneficial for broader, interdisciplinary calls like ‘Open Up’, where reviewers’ foundational understanding across fields could facilitate holistic and innovative assessment of proposals bridging traditional disciplinary boundaries.

**Fig. 13:**
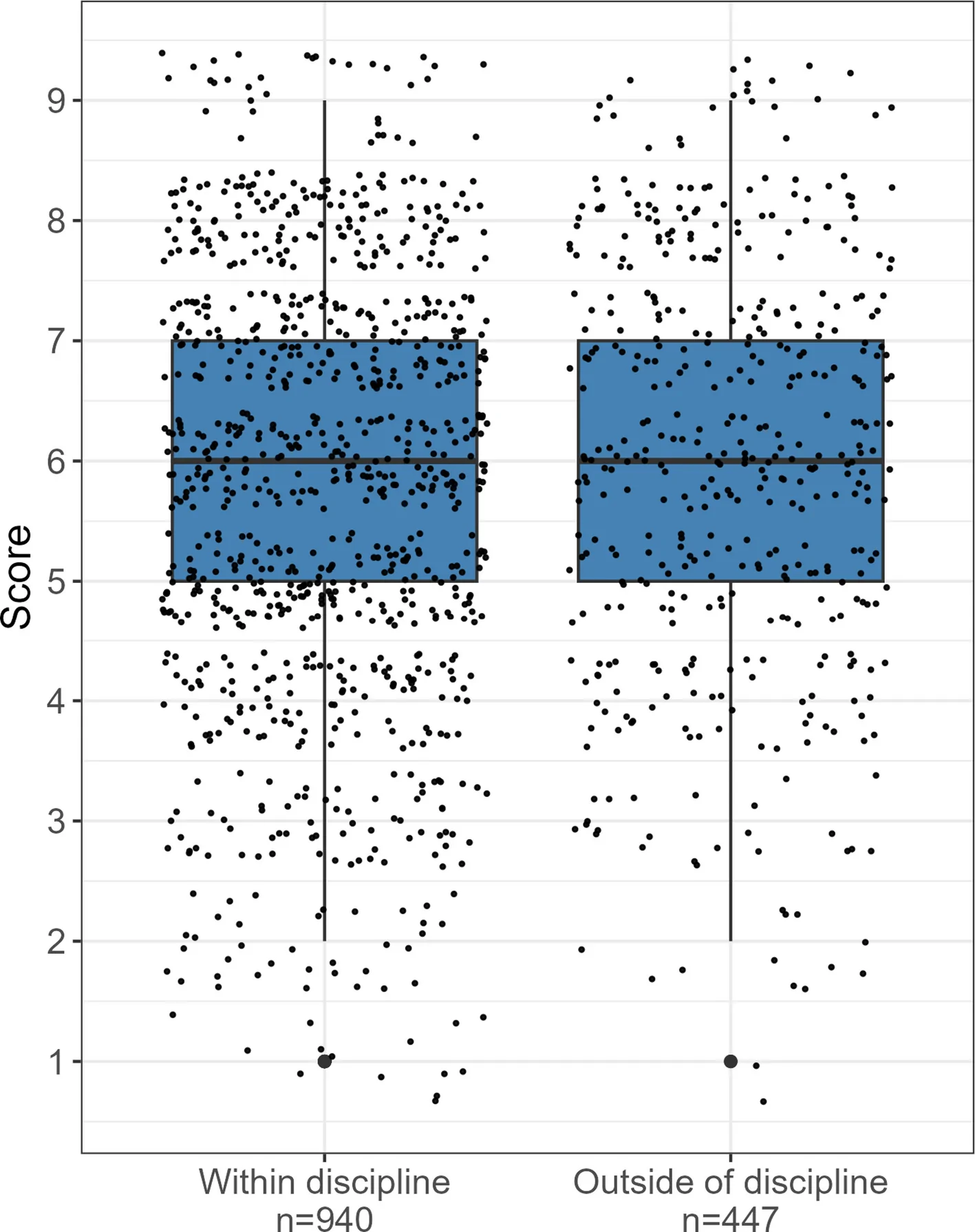
Score awarded according to disciplinary overlap

We found no evidence that outside of discipline reviewers disadvantaged proposals; differences in average scores based on disciplinary overlap were minimal (Fig. 13). In fact, outside of discipline reviewers awarded slightly higher scores (F(1, 1385) = 6.48, *p* = .011), suggesting homogeneity of the applicant pool is not a prerequisite for DPR, at least in interdisciplinary schemes. DPR may be more challenging for calls that are broad but lack an interdisciplinary focus.

### Managing the DPR process (RQ6)

Factors which should be considered in the implementation and management of a DPR process were identified in addition to mitigating measures (Table 5).

**Table 5:** Key considerations and potential mitigating measures in the management of a DPR process

Key consideration	Quotation	Mitigating measures
Initial effort/workload to implement	“It's double work. It's not only caring about the panel members and inviting them and arranging everything for this meeting and their reviews. It's also working with hundreds of people who will do the reviews and having contact with them and the group of people who are interested in ‘Open Up’…They want to discuss about it [and] they have lots of questions.” *(Barbara Neubauer, VWS staff)*	Leverage insights from previous DPR trials, e.g. [33]
Concerns about ‘gaming’	“The interest of the applicants consists in rating others lower than themselves, right? Because there is of course the interest in getting your own proposal funded. And I don't know how to work around that or whether it impacts the outcomes… this is something I was worried about.” *(P13, panel member)*	Transparent and consistent communication Mechanisms e.g. separate pools to prevent ‘successful’ gaming
Funder has less direct influence on process	“In the panel meeting you can just emphasise much more what the Foundation wants with the call… So there is a way to kind of remind the panels what this call is all about, which we experience is sometimes really necessary. This is corrective [and] this is of course lacking in the DPR.” *(P4, VWS staff)*	Funder retains right to veto decision Two-stage process

Funders may want to consider incorporating additional innovations alongside DPR. Applicants suggested various modifications, covered in detail in [35]. All results are summarised in Table 6.

**Table 6:** Summary and integration of qualitative and quantitative results

Research Question	Qualitative results	Quantitative results	Integration and evaluation of results	Meta-conclusion
RQ1 What were different stakeholders' expectations for the DPR experiment?	Applicants’ main concerns were being in competition with other reviewers, potential for gaming, time and workload burden and lack of reviewer expertise Panel members were open and positive about DPR but had some concerns re: quality of reviews; lack of discussion and applicant workload VWS staff were concerned re: burden on applicants, reduced applications and review quality/expertise	Over 70% applicants agreed DPR would select the best proposals, was fair, was suitable, would identify appropriate reviewers and would select more adventurous proposals Less than 50% of applicants agreed that DPR and panel review would select the same proposals	Depth and breadth of evidence: Qualitative results provide evidence across all stakeholder groups. Quantitative results provide evidence from all applicants Qualitative and quantitative results were consistent Qualitative results may explain why a proportion of applicants had reservations about DPR	Expectations of DPR were relatively positive though there were also some concerns. Concerns common to all stakeholder groups included the burden on applicants, reviewer expertise/review quality
RQ2 What are the main points of comparison between DPR and the classic panel process?	The focus of DPR reviewers and panel members may have differed. Panellists may have paid greater attention to overall quality and DPR to originality of the idea Nature of decision making differed, DPR relies on individual judgements whilst the panel review process also includes collaborative, group decision-making	Three proposals were selected by both processes Nine out of ten DPR funded proposals were shortlisted in the panel process. Eight out of ten DPR funded proposals were discussed during the panel meeting Across the 70 proposals reviewed in both processes, simulated rankings revealed modest agreement between DPR and panel reviewers, this was greatest for the proposals in the top quartile (0.39) Criteria scores strongly predicted overall scores in both processes. Reviewers in both processes used criteria similarly; greatest weight was given to originality, least to added value of the constellation	Quantitative results quantified and tested the extent of agreement between the processes (where possible) and in the overall funding decision Qualitative results provided insight into the nature of decision making, a more nuanced understanding of the similarities and differences in processes, and potential causes of divergence	There was some overlap between DPR and the panel review process but also some divergence, including in funding decisions. Neither DPR nor the panel review process decisions offer a ground truth, subjectivity is inherent in both processes Similarities (e.g. emphasis on criteria in reviews) and differences (individual decision-making in DPR vs individual and group decision-making in the panel process) in the nature of decision-making were evident between the two processes
RQ3 How reliable/robust is the evaluation conducted using DPR?	N/A	Across 500 bootstrapped samples a mean of 5.49 (SD = 1.29) DPR funded proposals would still be funded Across all proposals, across all samples: - 74.3% were sometimes funded - 25.7% were never funded - No proposal was always funded Some uncertainty was reported for most review decisions (M = 2.04, SD = 1.19) No significant association between average proposal uncertainty and final rank F(1,138) = 2.93, *p* =.089, R^2^ = 0.02; ꞵ = 15.68 (95% CI[−2.42, 33.78]) Across all DPR reviews: more variance was attributable to differences between reviewers (20.0%) than differences between proposals (8.9%) Excluding trimmed reviews: more variance attributable to differences between proposals (19.4%) than differences between reviewers (9.2%) DPR reviewers appear consistent in their behaviour within reviews; criteria scores strongly predicted overall score	Evidence for this research question came from the quantitative stream only	In DPR, the number of reviews per proposal means stability and variability can be evaluated and quantified. Modest stability of rankings/funding decisions may be explained by inter-reviewer variability. Trimming improves but does not resolve this
RQ4 How did different stakeholders perceive DPR after seeing it in practice?	Potential for shorter overall decision time is appreciated by all stakeholders. Burden for applicants remained a concern but appeared manageable due to shorter proposals Applicants appreciated receiving more, and more diverse, feedback from ‘true’ peers. Some felt the DPR was fairer and more democratic DPR can be a valuable learning experience, e.g. reviewing and grant writing which benefits funders and researchers Lack of discursive component in DPR reduces influence of group/social dynamics but also opportunity for clarification Solved difficulties in reviewer recruitment for funder but, as a new process, required time/resources to implement and manage Potential for gaming and lack of reviewer expertise remained a concern. Scooping was not highlighted as a concern by applicants	Early and later career researchers reported more mixed attitudes about taking part in future funding calls using DPR Most proposals received more attention under DPR (Med = 12.33 h) than the panel process (45 min- 4 h) In DPR, each reviewer spent a median of 4 h reviewing all allocated proposals. Panel members spent almost 2.5 times longer (Med = 9.92 h) completing peer reviews DPR reviewing took place over ~6 weeks, panel review process activities took ~13–14 weeks Statistically indistinguishable self-rated disciplinary fit between DPR (M = 4.92, SE = 0.03) and panel (M = 5.02, SE = 0.11) reviewers 91.6% of applicants considered reviews polite and 69.7% considered them helpful. 51% considered their received reviews’ expertise to be good 63.4% of reviews were considered constructive and 22.3% non-constructive Agreement re constructiveness of comments varied between co-applicants and on average was only ‘fair’ (weighted κ = 0.38)	Qualitative stream provides evidence re perceived advantages and disadvantages across all stakeholder groups Quantitative provides evidence of perceptions from around ⅓ of applicants (n = 123). Other relevant evidence, e.g. time taken to review, includes all applicants/is at the study level Some factors were only apparent in one set of results. The quantitative highlighted potential advantages to applicants which individual applicants would not have insight into The qualitative stream highlighted potential advantages which cannot be measured, or which have outcomes which it is too early to measure Evidence is broadly consistent between the two streams. Some perceptions are not supported by other evidence e.g. perceptions of reviewer expertise (qualitative and quantitative) and evidence of the influence of this on outcomes	Several advantages and disadvantages of DPR were identified by different stakeholders. Attitudes to DPR were more mixed in some groups including ECRs The concurrent nature of reviewing under DPR means that DPR has the potential to offer faster time-to-decision. Sharing reviewing responsibilities more widely reduces the reviewing undertaken by any individual but increases the workload for applicants Concerns regarding reviewer expertise remained, though there is limited empirical evidence to suggest this influenced reviewing or funding decisions
RQ5 When and where is DPR most, and least, suitable for use?	No consensus as to when DPR is most suitable. Some suggested it was more appropriate for more homogenous disciplines or applicants whilst others suggested better for broader, interdisciplinary calls Suggestions that it may be more suited to innovative, creative, nontraditional funding schemes but no consensus as to whether innovative/novel projects are favoured	Minimal influence of disciplinary overlap on review scores (F(1, 1385) = 6.48, *p* =.011) DPR is scalable; there is no upper limit to the number of applications/applicants who can be involved	Quantitative evidence partially resolves the lack of consensus in the qualitative evidence regarding when DPR is most suitable Qualitative stream provides suggestions from stakeholders as to specific types of schemes in which DPR might be more acceptable to applicants	Evidence from this trial of DPR does not preclude use of DPR in broader, interdisciplinary calls with less homogenous applicants Factors including type of applicant, discipline, career stage and openness to innovative approaches may influence acceptability
RQ6 What are the key management challenges in managing a DPR process?	Workload for funders- initially high but once established the process is replicable Managing or mitigating gaming/strategic behaviour Funders may want to retain ultimate power of veto; less influence on process when DPR is used	Increasing the number of reviews per proposal increases stability (mean no. DPR funded proposals still funded) but there is no optimal number A small number of reviewers gave scores consistently below the proposal mean. These scores were almost always removed by trimming	The qualitative stream identifies some of the key challenges of managing DPR processes. The quantitative stream offers some evidence for some of these challenges	Funders are required to make tradeoffs across various aspects of the DPR process. E.g. stability/applicant burden, retaining veto/democratisation etc Transparency and communication throughout the process may deter, and allay concerns about, gaming

## Discussion

Expectations of DPR were generally positive, though stakeholders also shared concerns (RQ1). Those specific to DPR (e.g., potential for ‘gaming’) reflect views reported elsewhere [24], but this experiment provided evidence in the particular context of funding allocation. Some observations reflected more general concerns regarding grant evaluation (e.g., reviewers lacking relevant expertise) [3, 9, 14]. DPR’s novelty may amplify concerns without necessarily increasing risk of negative outcomes over conventional processes.

We observed some overlap across both processes and funding decisions (RQ2). Greater agreement between the two systems at earlier stages aligns with previous work suggesting that panel discussion increases differences in terms of decisions between the two systems [22, 31]. It also provides some support for the notion that the discursive element of panel review and group decision-making it entails, perceived as critical by some participants, adds to the process, enabling clarification and encouraging the incorporation of multiple and diverse perspectives. Whilst DPR and the panel process ultimately selected (mostly) different proposals, the two processes were differently structured and not symmetrical. Divergence between the systems, with selection occurring at both ends of the panel process, means that the extent to which they disagreed is less clear. At present, comparison of outcomes of the projects selected for funding via each process is not possible. When funded projects mature, and as DPR is more widely adopted, it will be possible, and essential, to empirically examine outcomes.

Stability of funding decisions in DPR was modest, likely due to high inter-reviewer variability (RQ3). Low levels of agreement between reviewers evaluating the same proposals is a common issue in grant peer review [4, 5]. Whilst trimming scores reduces the variance attributable to inter-reviewer differences it does not resolve this issue. It is also just one of many possible approaches to score aggregation including use of untrimmed means, median scores, reviewer-standardisation, or weighting scores according to confidence. Alternative approaches such as reviewer-standardisation may limit the influence of between-reviewer differences but raises other issues including arbitrary decisions about approach to calibration, loss of meaningful variation, and diminished explainability. Reviewer uncertainty, whilst also common, may have contributed to the observed variability [44]. Increasing the number of reviews per proposal increased stability to an extent, although no number of reviews would produce completely consistent results.

DPR was understood to afford advantages and disadvantages for applicants and funders (RQ4). Both benefit from increased efficiency, faster decision times, and democratisation of the review process. Increased burden on applicants, loss of the discursive element of the panel review process, and perceived lack of reviewer expertise were noted disadvantages. Indeed, applicant concerns regarding reviewer expertise persisted and, potentially, increased during the process. This may represent a genuine belief that reviews lacked expertise, a well-documented concern in DPR and peer review more broadly [24, 25]. Alternatively, it may reflect an overattribution based on an applicant’s own self-perceived lack of expertise. There was, of course, no comparator since, per the standard ‘Open Up’ panel review process procedure, panel feedback comments were not shared with applicants. In any case, whilst often equated with disciplinary alignment [19, 45], ‘expertise’ is a multi-dimensional and variable concept [46]. Some applicants may have perceived it as synonymous with experience, viewing the inclusion of all applicants, including novices, as reducing the expertise of the reviewer pool.

Attitudes toward DPR were more mixed among earlier and later career researchers; muted positivity for the former may be due to the added burden of reviewing given precarious employment, while for the latter it may reflect their established role as senior gatekeepers.

Previous DPR trials have tended toward funding programmes with relatively homogenous applicants and topics [22, 24, 26, 28]. This experiment demonstrates the applicability of DPR to a broad, innovation-focussed interdisciplinary funding call at a large, private funding foundation (RQ5). While interviewees lacked consensus on whether DPR was better suited for broad or narrow calls, our quantitative findings do not preclude its use in interdisciplinary schemes with less homogenous applicants. Greater alignment in expertise may actually result in lower scores being awarded [11, 45], as was marginally seen here. Domain experts may also prioritise feasibility over novelty [11, 47], meaning for calls such as this, where innovation is the focus, the heterogeneity of the applicant and reviewer pool could actually be advantageous.

Implementing DPR requires funders to consider several necessary trade-offs (RQ6). Increasing the number of required reviews enhances the stability of funding decisions but increases applicant burden. Diversifying the reviewer pool may introduce greater variation in perceived expertise and/or experience. Democratising funding allocation requires funders to cede some control over funding decisions to the wider research community.

DPR represents a fundamental shift in the way peer review and decision-making are conducted: *from the power of the gatekeeper to the wisdom of the (expert) crowd*. Traditional funding decision making valorises senior gatekeepers whose experience is paramount, despite their possibly limited in-depth expertise across proposals. Discussion between gatekeepers has been seen as a valuable part of the decision-making process, helping to identify the best outcomes [48]. DPR, conversely, places greater emphasis on collective decision-making involving researchers at different career stages.

Communication is a second overarching consideration for DPR implementation. Transparency about the motivation for using DPR may build trust between funder and applicants [3, 49] and enhance openness to change [50, 51]. Funders should explicitly acknowledge and address potential applicant concerns, though anticipated concerns may not actually materialise; in line with previous work [22, 24], we found issues of confidentiality and scooping were not considered any more problematic than in conventional review. Discrepancies between perceived and actual concerns highlight the need for better understanding of the research community’s attitude to innovation in funding evaluation.

Communication can also augment more direct intervention. Organisations should consider methods to prevent “gaming” from influencing outcomes, such as partitioning, auditing scores, or rewarding honesty [52]. Carefully framed communications are crucial to reinforce these efforts by discouraging *attempts* at “gaming” (whether success is possible or not), which may still disrupt the process.

Finally, specificity in communications helps maximise consistency. Providing clear and specific criteria and instructions may limit the impact of reviewer subjectivity [53] and reduce “commensuration bias” (reviewers prioritising different aspects of the proposal or judgement criteria) [54]. Offering brief training may increase consistency [55] and further DPR’s suggested training and learning benefits.

DPR and the panel process did not produce identical results; only three out of 18 funded proposals were selected by both mechanisms. Whilst anticipated by some—less than half of applicants believed the two processes would select a similar set of proposals—we appreciate that for others this may be a source of concern. Assessing and certifying quality is, of course, an important function of peer review, though others have also been identified [56, 57]. Instinctively we may therefore expect that both processes should select the same proposals, i.e. those which are of highest quality. Considering the diverse factors that influence both this assessment, e.g. individual notions of quality, differential emphasis of criteria etc., and the overall funding decision, e.g. where the funding line is drawn, method of aggregating scores/determining the final decision etc., provides some context to this divergence. Indeed, prior work demonstrates that even the same funding system with different actors may produce different outcomes [4]. Innovative approaches that address recognised problems in conventional systems should also expect, and perhaps welcome, different outcomes, rather than viewing them as problematic. Implementing DPR, or any innovation in funding evaluation, represents a shift from the status quo. It requires stakeholders to move beyond judging a new system by its ability to reproduce outcomes of conventional systems.

Our findings reflect the subjectivity inherent in peer review, evidenced by high inter-reviewer variability, reviewer uncertainty, and inconsistent ratings of review constructiveness. Modifying the process – for example, ranking rather than rating proposals to address scoring miscalibration [58] – may reduce variability. However, our analysis suggests inter-reviewer differences cannot simply be averaged out to achieve certainty – there is irreducible uncertainty beyond different reviewers being more or less generous in their scoring [25, 59], or scores being affected by random noise, and which probably reflects “commensuration bias” [54]. We echo previous calls to acknowledge, measure, and evaluate this uncertainty [9]**.**

Of course, peer review will always involve subjectivities; perfectly objective peer review is unobtainable, whatever the system used, since knowledge itself is situated and perspectival. For the interdisciplinary and innovation-focused call studied, a variety of perspectives was particularly likely. DPR enhances the visibility of these subjectivities, moving the process out of the conventional "black box" that traditional peer review often is (P9). While advocates see this transparency as positive, it creates risks, including greater applicant dissatisfaction. In the current study, when applicants express unhappiness with a review produced by DPR, it is important to note that not only could they not make comparisons with the panel process, since in the latter materials were not shared, but also that at least some of the concerns raised are not unique to DPR and are inherent within traditional funding evaluation systems; they are just not publicly visible.

### Strengths

A key strength of this paper is its comparison of DPR and a conventional funding evaluation process; it is the first trial to implement parallel evaluation using both methods on the same proposals. Furthermore, this study demonstrates the feasibility of DPR within an interdisciplinary scheme in the Social Sciences and Humanities for the first time. Our mixed-methods approach provides a comprehensive overview that fully incorporates the perspectives of multiple stakeholders.

### Limitations

The current study demonstrates the feasibility and potential of DPR as a final decision-making and feedback mechanism in an interdisciplinary, innovation-focussed funding call at a private funder. These results may not be generalisable to different types of funding calls or alternative applications of DPR, e.g. for screening.

Both interviews and the feedback survey were completed after funding decisions were communicated. Funded applicants were overrepresented in both, so applicant experiences and perceptions of DPR and the reviews received may have been influenced by funding success or lack thereof. Recollections of expectations of DPR and earlier stages of the process may be subject to recall bias. Sample size for quantitative analyses was determined by the number of proposals submitted and reviews completed. Where unit of analysis was at the proposal level, statistical power may have been limited.

Our measure of disciplinary overlap, whilst consistent with the definition used in the proposal-reviewer matching algorithm, may under or overestimate true disciplinary fit. The measure of reviewer uncertainty also has limitations. It may have been affected by scale interpretation or scoring habits or restricted by the level of the overall score awarded.

Finally, our evaluation extended only to receipt of funding decisions; we could not examine the outcomes of funded proposals (e.g., project success, impact). There is a crucial need for ongoing, long-term research to track the outcomes of funded projects to fully determine the preferable selection methods.

## Conclusions

This study demonstrates the feasibility and practical requirements for implementing DPR as a decision-making and feedback mechanism in grant evaluation for an interdisciplinary, innovation-focussed funding programme. Due to the number of reviews per proposal, DPR allows novel insight into the nature of peer review. Divergence between DPR and panel review processes and funding decisions, whilst not unexpected, can challenge assumptions about grant proposal evaluation. It also reinforces the importance of evaluating innovative approaches by more than their capacity to reproduce outcomes of traditional funding mechanisms. Our interviews with stakeholders reveal the crucial trade-offs funders face in designing selection mechanisms and highlight the essential acceptability of funder innovation within the research community. We show that DPR can shorten the time for grant evaluation and funding decisions, whilst simultaneously providing constructive feedback to all applicants. Overall, the trial underscores the potential of DPR as an innovation which builds on the established principle of peer review but deploys it in a more “democratic” rather than “gatekeeping” mode.

## Supplementary Information

Supplementary Material 1.

## Data Availability

The anonymised qualitative dataset generated and analysed during the current study is available at https://doi.org/10.17605/OSF.IO/WMQPH. Anonymised quantitative data from the DPR arm of the experiment is available at https://doi.org/10.17605/OSF.IO/YF697.

## Abbreviations

DPR: Distributed peer review
ECR: Early career researcher
ICC: Intraclass correlation coefficient
IQR: Interquartile range
NSF: National Science Foundation
SD: Standard deviation
SE: Standard error
UKRI: UK Research and Innovation
VWS: Volkswagen Foundation
