# Supplementary material for: Applicants as reviewers: a mixed methods evaluation of the risks, benefits, and potential of distributed peer review for grant funding allocations
Source: Res Integr Peer Rev. 2026 Sep 12;11:54. doi: 10.1186/s41073-026-00246-7 (PMC13617845; doi:10.1186/s41073-026-00246-7)
Supplement: Supplementary file 1 [file 41073_2026_246_MOESM1_ESM.docx]

**Supplementary Material**

**Contents**

**Open Up funding call description**………………………………………………………………..1

**Open Up proposal template**……………………………………………………………………….2

**Participant consent information**………………………………………………………………….2

**Supplementary Table 1:** Characteristics of applicants and applicants who responded to the feedback questionnaire………………………………………………………………………………4

**Supplementary Table 2:** Codebook for qualitative interviews………………………..…………5

**Supplementary Figure 1:** Distribution of DPR reviewer uncertainty for all review decisions (N=1387)……………………………………………………………………………………………….8

**Supplementary Table 3:** Sensitivity analyses for bootstrap stability analyses …….…………8

**Supplementary Table 4:** Model fit and likelihood ratio tests for mixed effects model examining random effects of proposal and reviewer on score awarded………………………………….....9

**Supplementary Table 5:** Constructiveness of review comments………………………..……10

**Open Up funding call description**

Description of ‘Open Up’ as shared on Volkswagen Foundation website and in public facing materials.

Open Up – New Research Spaces for the Humanities and Cultural Studies

Profile area: Exploration

“To 'open up' something is associated with the curiosity to take the first step into something new and unknown. In this sense, we are looking for projects that not only offer new perspectives on already known research subjects, but also explore entirely new research spaces and topics.

The funding offer assumes that these topics are currently characterized by a particular complexity and require a multi-perspective approach in order to reveal and understand this complexity. We therefore aim to support researchers who open up new areas of research and knowledge in the humanities and cultural studies by jointly exploring complex and hitherto unexplored research topics. By funding collaborations between two or three researchers, the Foundation intends to facilitate the full creative potential of the interaction of different expertise and perspectives.”

**‘Open Up’ proposal template**

Open Up – New Research Spaces for the Humanities and Cultural Studies

Please complete without specifying author, institute or career level.

Title of the project:

Proposals are assigned to reviewers according to the DFG subject area structure. Please select at least one and up to three subject areas that you can associate with your proposal. If you cannot assign your proposal to any of the disciplines in the drop-down menu, please use the text box. Please also refer to the DFG subject area structure when using the text box.

1 Exploratory research question (max. 30 words)

2 Project description (max. 500 words)

3 Scientific relevance and novelty (max. 500 words)

4 Added value of the constellation (max. 250 words)

5 References

**Participant consent information**

Applications to the call “Open Up – New Research Spaces for the Humanities and Cultural Studies” will be reviewed by the conventional Panel Review Process (PRP) and also by a Distributed Peer Review process (DPR). Proposals selected by either mechanism will be funded. The process and outcomes will be formally evaluated to allow for comparison and to contribute evidence on how research funding can be made more efficient, effective and equitable. More information on the call, on Distributed Peer Review and the evaluation of this process is provided on the Open Up Website.

Benefits

The experiment provides each applicant with two opportunities to receive funding – via the conventional panel review and via the distributed peer review mechanism. Otherwise, there are no direct benefits, although participation supports evidence-informed innovation in peer review and funding mechanisms.

Data management

Data from this project exists in three categories:

• Proposal data – data collected as a normal part of the funding call process. This

includes proposals, CVs, contact details of applicants and co-applicants. This will be

managed and stored by the Volkswagen Foundation according to their standard

procedures. Anonymised grant proposals will be shared for review with selected other applicants.

• Panel data – data collected as a normal part of the funding call process. This includes panel reviews and funding decisions. This will be managed and stored by the

Volkswagen Foundation according to their standard procedures. Quantitative and

evaluation measures such as review scores, post-panel scoring and funding outcome

will be anonymised, aligned with and added to the review data.

• Review data – data collected as part of the distributed peer review process, and its

evaluation. This includes scoring of proposals and associated survey questions. This will be anonymised and kept separate from the proposal data. It will be managed and stored by the Volkswagen Foundation. The Research Team will have access. No identifying details of any applicants will be shared with the Research Team.

All data, regardless of type, will be held by the Volkswagen Foundation and shared with the Research Team only to the extent necessary according to the purposes below. Personal data will only be accessed by the Volkswagen Foundation.

Post project, quantitative and evaluation measures – but not data with risk of deanonymisation (such as a review text comments) – may be archived in a public research data repository by the Volkswagen Foundation.

Please take note of the following information

By submitting to the call “Open Up – New Research Spaces for the Humanities and Cultural Studies” you

• Agree to have your proposal evaluated by both the conventional Panel Review Process and the Distributed Peer Review process.

• Agree to participate in the Distributed Peer Review process by submitting reviews of the proposals you are requested to review by the Foundation.

• Understand that failure to submit reviews will result in your own proposal being

eliminated from consideration.

• Understand that your proposal and applicant data will be held by Volkswagen

Foundation in accordance with GDPR (see Data Protection Information for Applicants

and Grant Recipients).

• Understand that anonymised data on applications and reviews will be shared with

researchers from the Research on Research Institute and the University of Sheffield, for the purpose of analysis and evaluation of the review processes.

• Anonymised quantitative data (e.g. review scores) may be included in published reports, but no details which identify individual proposals, or applicants, or reviewers will be shared publicly.

• Agree that you may be contacted post-submission to be invited – with no obligation – to participate in interviews and reflection on the process. Information on the interview process will be provided separately to all those who agree to participate, who will also be asked separately to give their consent to take part in interviews.

Contact

For questions about the call or this agreement please contact:

Independent scientific advice and ethical review provided by the University of Sheffield, UK.

Please contact:

If you feel your complaint, concern or incident has not been handled in a satisfactory manner, contact:

**Supplementary Table 1:** Characteristics of applicants and applicants who responded to the feedback questionnaire

|  | **All applicants**  **(N= 323)** | | **Applicants providing feedback**  **(N =127)** | |
| --- | --- | --- | --- | --- |
| **Characteristic** | **No.** | **Percentage** | **No.** | **Percentage** |
| *Gender:*  Diverse | 3 | 0.93% | 3 | 2.36% |
| Female | 151 | 46.75% | 67 | 52.76% |
| Male | 160 | 49.54% | 55 | 43.31% |
| No answer | 9 | 2.79% | 2 | 1.57% |
| *First generation:*  Yes | 114 | 35.29% | 49 | 38.58% |
| No | 194 | 60.06% | 74 | 58.27% |
| I don't know | 4 | 1.24% | 1 | 0.79% |
| No answer | 11 | 3.41% | 3 | 2.36% |
| *Working years quantiles:*  0-8 | 89 | 27.55% | 35 | 27.56% |
| 9-12 | 72 | 22.29% | 24 | 18.90% |
| 13-19 | 80 | 24.77% | 35 | 27.56% |
| 20-52 | 81 | 25.08% | 33 | 25.98% |
| No answer | 1 | 0.31% | - | - |
|  | **Mean (SD)** | **Median (IQR)** | **Mean (SD)** | **Median (IQR)** |
| Working years | 14.47  (8.90) | 12.5  (11.75) | 14.39  (8.22) | 14  (12) |

**Supplementary Table 2:** Codebook for qualitative interviews

| Name | Files | References |
| --- | --- | --- |
| Alternatives or innovations in PR | 19 | 37 |
| Background | 1 | 1 |
| Benefits of DPR | 20 | 120 |
| Cheaper | 2 | 3 |
| Filters speculative applications | 1 | 1 |
| Innovative or interesting process | 11 | 18 |
| Less bias | 5 | 5 |
| Still potential for bias | 1 | 1 |
| More anonymity | 4 | 4 |
| More diverse feedback | 8 | 12 |
| More feedback | 7 | 16 |
| Promotes innovation | 5 | 5 |
| Reduced individual workload or boredom effect | 1 | 2 |
| Reviewer recruitment solved | 7 | 9 |
| Reviewers are more true peers | 9 | 10 |
| Reviewers more invested | 7 | 9 |
| Shorter overall decision time | 6 | 7 |
| Supports reviewer understanding of call criteria | 6 | 8 |
| Trains reviewers in reviewing and writing | 7 | 11 |
| Challenges of DPR | 19 | 130 |
| Anti-innovation | 5 | 10 |
| Award power is devolved | 1 | 2 |
| Burden on applicants | 15 | 29 |
| Burden on funder | 2 | 2 |
| Feeling overseen by funder | 1 | 2 |
| Gaming | 14 | 21 |
| Lack of reviewer expertise | 14 | 31 |
| Lack of specificity | 1 | 1 |
| Potential for bias | 6 | 9 |
| Review quality | 8 | 12 |
| Scooping | 4 | 4 |
| Subjectivity or unpredictability | 6 | 7 |
| Fairness | 19 | 31 |
| Feedback | 14 | 51 |
| Helpful or constructive | 11 | 24 |
| Neutral | 6 | 9 |
| Unhelpful or unconstructive | 9 | 16 |
| Future of DPR | 16 | 27 |
| Managing process issues around DPR | 8 | 19 |
| Overlap between DPR and panel | 8 | 15 |
| Panel process | 19 | 140 |
| Benefits of panel process | 15 | 57 |
| Ability to discuss | 8 | 18 |
| Can select 'best' reviewers | 4 | 5 |
| Established, works fine | 6 | 9 |
| Funder can veto | 1 | 3 |
| Increased funder interaction | 3 | 5 |
| More expertise or knowledge | 10 | 10 |
| Panel are better trained or more experienced in process | 6 | 6 |
| Challenges of panel process | 18 | 70 |
| Lack of diversity | 6 | 7 |
| Lack of expertise | 7 | 11 |
| Lack of feedback for applicants | 2 | 2 |
| Lack of knowledge or transparency about process | 4 | 4 |
| Personal dynamics | 10 | 19 |
| Potential for bias | 7 | 14 |
| Reviewer recruitment | 5 | 5 |
| Small number of reviewers | 5 | 8 |
| Expectations | 1 | 1 |
| Process of negotiation | 3 | 9 |
| Reviewing proposal vs idea | 3 | 3 |
| Proposal writing | 8 | 19 |
| Review criteria and guidelines | 18 | 44 |
| When to use DPR | 3 | 5 |

**Supplementary Figure 1:** Distribution of DPR reviewer uncertainty for all review decisions (N=1387)

**Supplementary Table 3:** Sensitivity analyses for bootstrap stability analyses

| **No bootstrapped samples** | **Mean no still funded (SD)** | **% Always funded** | **% Sometimes funded** | **% Never funded** |
| --- | --- | --- | --- | --- |
| 1000 | 5.51 (1.24) | 0% | 24.29% | 75.71% |
| 2000 | 5.52 (1.24) | 0% | 20% | 80% |
| 3000 | 5.52 (1.26) | 0% | 17.86% | 82.14% |

**Supplementary Table 4:** Model fit and likelihood ratio tests for mixed effects model examining random effects of proposal and reviewer on score awarded

|  | **All reviews**  **(N=1387)** | | | | | **Excluding trimmed reviews**  **(N=1107)** | | | | |
| --- | --- | --- | --- | --- | --- | --- | --- | --- | --- | --- |
|  | **Model fit** | | | **Likelihood ratio test**  **(Against unconditional model)** | | **Model fit** | | | **Likelihood ratio test**  **(Against unconditional model)** | |
| **Model** | **AIC** | **BIC** | **Log likelihood** | 𝜲^2^ | ***p*** | **AIC** | **BIC** | **Log likelihood** | 𝜲^2^ | ***p*** |
| Proposal (unconditional) | 5549.7 | 5565.5 | -2771.9 | - | - | 3919.1 | 3934.1 | -1956.5 | - | - |
| Proposal + Reviewer | 5471.7 | 5492.6 | -2731.8 | 80.10 | <.001 | 3907.4 | 3927.5 | -1949.7 | 13.69 | <.001 |
| Proposal + Reviewer * Disciplinary overlap | 5498.5 | 5519.4 | -2745.2 | 53.25 | <.001 | 3913.1 | 3933.2 | -1952.6 | 8.00 | .005 |

**Supplementary Table 5:** Constructiveness of review comments

| **Constructiveness of review comment** | **Count** | **Percentage** |
| --- | --- | --- |
| Constructive | 451 | 36.11% |
| Somewhat constructive | 341 | 27.30% |
| Neutral | 178 | 14.25% |
| Somewhat non-constructive | 146 | 11.69% |
| Non-constructive | 133 | 10.65% |
